# AI‐Assisted Drug Re‐Purposing for Human Liver Fibrosis

**DOI:** 10.1002/advs.202508751

**Published:** 2025-09-14

**Authors:** Yuan Guan, Lu Cui, Jakkapong Inchai, Zhuoqing Fang, Jacky Law, Alberto Alonzo Garcia Brito, Annalisa Pawlosky, Juraj Gottweis, Alexander Daryin, Artiom Myaskovsky, Lakshmi Ramakrishnan, Anil Palepu, Kavita Kulkarni, Wei‐Hung Weng, Zhuanfen Cheng, Vivek Natarajan, Alan Karthikesalingam, Keran Rong, Yunhan Xu, Tao Tu, Gary Peltz

**Affiliations:** ^1^ Department of Anesthesiology Pain and Perioperative Medicine Stanford University School of Medicine Stanford CA 94305 USA; ^2^ Department of Pathology Stanford University School of Medicine Stanford CA7Department of Pathology Stanford University School of Medicine Stanford CA 94305 USA; ^3^ Faculty of Medicine Chiang Mai University Chiang Mai 50200 Thailand; ^4^ Google Cloud Zürich 8050 Switzerland; ^5^ Arc Institute Palo Alto Palo Alto CA 94304 USA; ^6^ Google Research Mountain View CA 94043 USA; ^7^ Google DeepMind Mountain View CA 94043 USA

**Keywords:** artificial Intelligence, hepatic Organoids, liver fibrosis

## Abstract

Liver fibrosis has few treatment options due to the poor quality of the available animal and in vitro models. To address this, a hypothesis generating multi‐agent AI system (AI co‐scientist) is used to assist in re‐purposing drugs for treatment of liver fibrosis and direct their experimental characterization. The anti‐fibrotic efficacy and toxicity of 25 drugs are serially assessed in multi‐lineage human hepatic organoids grown in microwells (i.e., microHOs). Remarkably, three previously characterized anti‐fibrotic drugs and two AI co‐scientist‐recommended drugs that targeted epigenomic modifiers exhibited significant anti‐fibrotic activity and they promoted liver regeneration. Analysis of these five anti‐fibrotic drugs revealed that they all can reduce the generation of activated myofibroblasts and that each drug have unique effects on mesenchymal cells that generated their anti‐fibrotic effects. Since all five of the anti‐fibrotic drugs reduced TGFβ‐induced chromatin structural changes, epigenomic changes play an important role in the pathogenesis of liver fibrosis. One AI co‐scientist recommended drug is an FDA‐approved anti‐cancer treatment (Vorinostat) that reduced TGFβ‐induced chromatin structural changes by 91% and promoted liver parenchymal cell regeneration in microHOs. Hence, the integrated use of AI co‐scientist and this microHO platform identified a potential new generation of liver fibrosis treatments that also promote liver regeneration.

## Graphical abstract

A multi‐agent AI (AI co‐scientist) is used to repurpose drugs for liver fibrosis treatment and to guide their experimental characterization. The anti‐fibrotic efficacy and toxicity of these drugs is assessed in multi‐lineage human hepatic organoids grown in microwells (microHOs). This approach identified new liver fibrosis treatments that also promote liver regeneration, offering hope for improved therapeutic options.

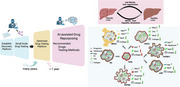

## 1 Introduction

Liver fibrosis is caused by extracellular matrix (**ECM**) accumulation in response to chronic liver injury, which is often caused by viral infection, non‐alcoholic steatohepatitis, or chronic alcohol exposure.^[^ ^1^ ^]^ This fibrotic state results from an interaction between parenchymal and nonparenchymal liver cells and possibly involves infiltrating immune cells.^[^ ^2^ ^]^ The key non‐parenchymal cells are the myofibroblasts (**MyoF**), which are generated in response to fibrogenic stimuli (especially TGFβ1) and they produce excess fibril‐forming collagens and other ECM proteins.^[^ ^2^, ^3^ ^]^ Irrespective of the inciting cause, activated MyoF and the ECM proteins they produce drive all forms of liver fibrosis. Patient outcome is determined by the extent of liver fibrosis,^[^ ^4^ ^]^ which is a major global cause of death (∼1M per year).^[^ ^5^ ^]^ Although drugs are being studied,^[^ ^1^, ^6^ ^]^ many agents that generated promising results in preclinical studies have failed in clinical trials due to safety concerns or lack of efficacy. The poor quality of available model systems has been the major barrier to identifying anti‐fibrotic therapies. Prior in vitro models did not have the spectrum of cell types that mediate fibrogenesis nor could they reproduce its key feature (i.e., production of thick collagen filaments^[^ ^7^ ^]^). Conclusions drawn from animal models are limited by concerns about species‐specific differences in fibrotic mechanisms.^[^ ^8^ ^]^ Hence, there are limited treatment options (other than when the underlying cause can be treated) for this prevalent and severe disease.^[^ ^9^ ^]^

Here, we use a pioneering approach to identify and characterize new anti‐fibrotic agents. First, we investigate whether a hypothesis‐generating multi‐agent AI system built with Gemini 2.0 (AI co‐scientist^[^ ^10^ ^]^) could identify anti‐fibrotic agents that target epigenomic modifiers, and if it could direct their experimental characterization. AI co‐scientist (details in Note S1, Supporting Information) utilizes a set of specialized agents for hypothesis generation, and experimental planning uses a “scientist in the loop” paradigm. Second, to examine the anti‐fibrotic efficacy of drugs suggested by AI co‐scientist we use an enhanced live cell imaging system that enables anti‐fibrotic efficacy and drug toxicity to be simultaneously assessed in multi‐lineage human hepatic organoids grown in microwells (i.e., “***microHOs***”).^[^ ^11^ ^]^ microHOs have hepatocytes, cholangiocytes, bile ducts, liver lobule architecture; and thick collagen filaments and MyoF appear in microHOs after TGFβ exposure. microHOs were used to perform the experiments suggested by AI co‐scientist to investigate the mechanism of action of five anti‐fibrotic agents and to characterize their effects on MyoF, mesenchymal cells and parenchymal cells.

## 2 Results

### 2.1 An Enhanced MicroHO Platform for Discovery and Characterization of Anti‐Fibrotic Drugs

**Figure 1:**
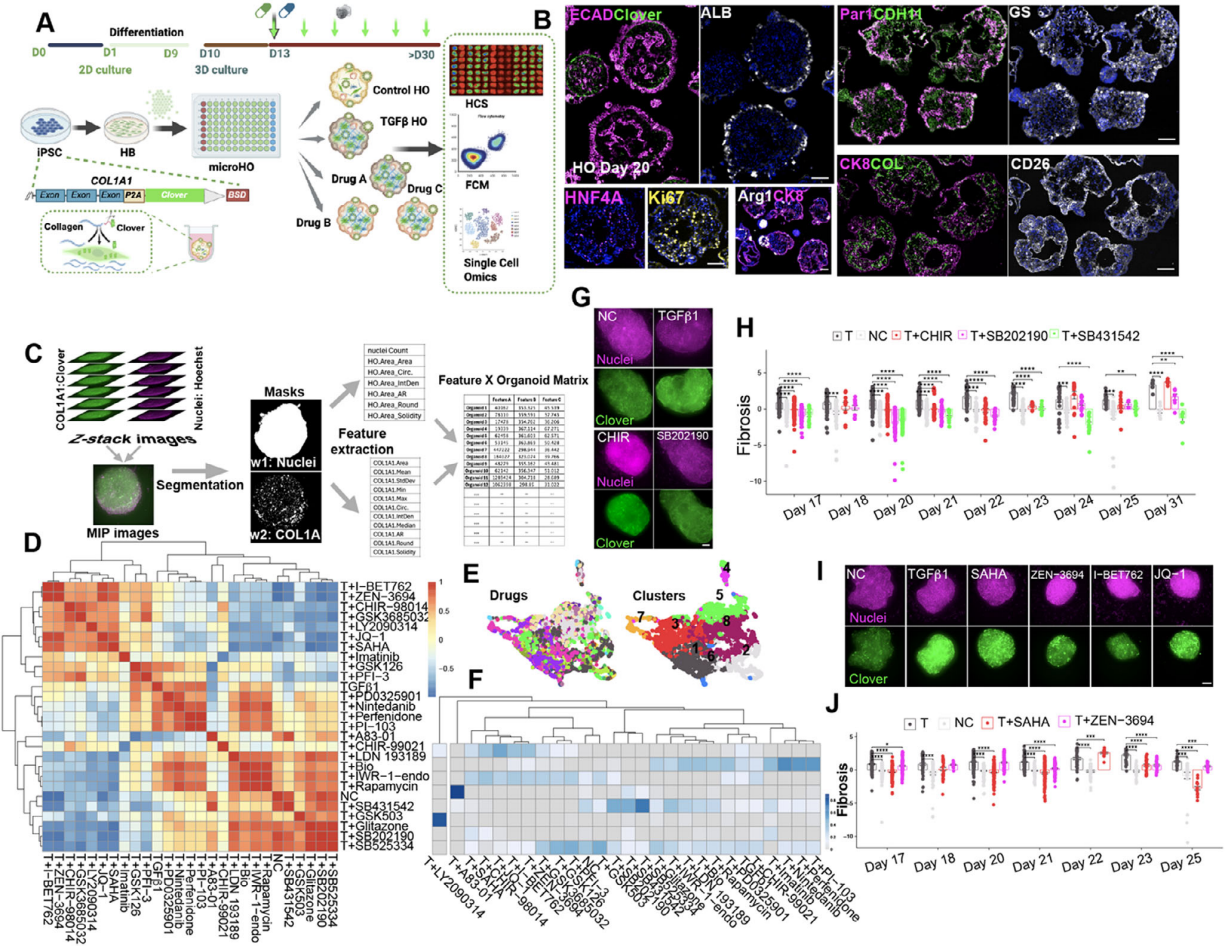
The microHO platform for assessing drug efficacy for treating hepatic fibrosis. A) A diagram of the microHO drug screening platform. An iPSC line has a P2A‐Clover insert at the 3′ end of *COL1A1*. The self‐cleaving P2A peptide enables *COL1A1* expressing cells in a microHO to be labelled with a fluorescent intracellular protein (Clover). Engineered COL1A1 reporter iPSCs are differentiated into hepatoblasts (HB), and 10000 HBs are placed in each microwell on day 10. TGFβ1 ± drugs are added on day 13, and the cultures are further differentiated into hepatic organoids. High content imaging is used to serially measure the fluorescence and cell number in microHOs. At the end of each experiment, samples for flow cytometry (FCM), scRNA‐seq and snATAC‐seq analyses are collected. B). Immunostaining of day 21 microHOs. microHOs using antibodies to ECAD, ALB, Par1, CDH1, GS, CK8, CD26, HNF4A, Ki67, Arg1, Clover, and COL1A. Scale bar is 100 µm. C). A diagram of the supervised imaging analysis workflow for microHOs. (i) Twenty to forty Z‐stacked images per microHO were prepared from the two channels used to analyze the extent of fibrosis (COL1A1:Clover) and cell number (number of nuclei labeled by the Hoechst 33 342 stain) for each microHO. (ii) Masks were prepared by region of interest (ROI) segmentation from each channel from the maximum intensity projection (MIP) image. (iii) Then, 18 features from the two channels were extracted to build the “Feature X organoid” matrix. **D**) A heatmap is generated from analysis of the *Pearson's* correlation coefficients calculated from mean values of 18 features measured in normal control (NC), TGFβ and TGFβ plus other 25 drugs treated microHOs. As shown here, the relative position of microHOs treated with TGFRB, GSK3β, p38 or HDAC (SAHA) inhibitors have the most similarity with NC microHOs. The TGFβ‐treated microHOs are very different from NC microHOs; and microHOs treated with EZH2, DNMT1, or PPARγ inhibitors have the least similarity with NC microHOs. The BRD4 inhibitor (Zen‐3964, I‐BET762, JQ‐1) effects cluster together and are intermediate between NC and TGFβ‐treated microHOs. The bar on the right shows the extent of the correlation (by color) for each square in the diagram: green (or magenta) indicates a positive (or negative) correlation. E) UMAPs show imaging data obtained from each of 16333 microHOs that are color coded based upon the treatment they received (left) or the clusters formed by k‐mean analysis (right). This data shows that the clusters in microHOs are differentially distributed based upon drug treatment. F) A heatmap is generated from analysis of the *Pearson's* correlation coefficients calculated from percentage of each cluster in microHOs treated with the indicated drugs. This data shows that microHOs treated with different drugs can be classified into different groups based upon the imaging features. G) Representative MIP images generated from day 21 microHOs treated with normal media (NC), 50 ng mL^−1^ TGFβ1, or TGFβ1 and drugs (CHIR‐99021, 3 µm or SB202190, 10 µm). Scale bar, 100 µm. H) The extent of fibrosis (COL1A1 fluorescence) was serially measured using the organoids in (G) on days 17 through 31. Each time point uses > 3 batches and each batch has > 8 microHOs. (ns, not significant; *p‐value < 0.05; **p‐value < 0.001; ***p‐value < 0.001; and ****p‐value < 0.0001). I) Representative images generated from day 21 microHOs treated with normal media (NC), 50 ng mL^−1^ TGFβ1, or TGFβ1 and drugs (SAHA, 1 µm; ZEN‐3694, 2 µm; I‐BET762, 2 µm; or JQ‐1, 1 µm). Scale bar, 100 µm. J) The extent of fibrosis (COL1A1 fluorescence) was serially measured using the microHOs in (I) on days 17 through 25. At least 3 experimental batches, which had >30 microHOs per treatment were assessed per condition per time point. The fibrosis and cell number measurements were normalized relative to NC microHOs. The p‐values are as in (H).

Our previously described microHO system^[^ ^12^ ^]^ and a high content imaging system were used to serially measure the antifibrotic effect of a drug. The collagen‐producing cells in microHOs are labeled with a fluorescent intracellular protein, which enables anti‐fibrotic efficacy to be serially analyzed. Drugs were added at the same time as a pro‐fibrotic stimulus (TGFβ), and the extent of fibrosis developing while the cells differentiate to form a multi‐lineage microHO was measured. Addition of a nuclear stain (Hoechst 33 342) enabled drug toxicity and antifibrotic efficacy to be simultaneously assessed. Samples for flow cytometry (FCM) and single‐cell RNA sequence (scRNA‐seq) analysis were collected at the endpoint of each experiment to characterize drug effects on specific cell types (**Figure** **1**A). Immunostaining indicates that microHOs resemble our previously characterized HOs;^[^ ^11^, ^13^ ^]^ they express liver parenchymal epithelial (ECAD, ALB, Par1, CK8, CD26, and HNF4A), mesenchymal cell (COL1A1::Clover, CDH11, and COL), proliferative (Ki67) and liver function (GS and Arg1) markers (Figure 1B).

A supervised image analysis workflow was used to analyze the twenty to forty Z‐stacked images collect from each microHO per time point. Information obtained from the nuclei and fibrosis channels were used to generate an 18 component “feature X organoid” matrix, which enhanced our ability to compare the effect of different drugs (Figure 1C). A scale function was used to normalize the raw measurements (z‐score) for each feature (fluorescence, size or nuclei number) and matrix parameters were developed from analysis of inter‐treatment variation across thousands of microHOs. To select matrix features, the data distribution and variance of each feature were plotted to select those whose variation in response to the many different treatments were above a threshold (total fluorescence (IntDen), nuclei count, etc.) (Figure S1A–C, Supporting Information). Pearson's correlation coefficients calculated for each matrix feature were used to generate a heatmap that enabled the anti‐fibrotic efficacy and cellular toxicity of 25 analyzed drugs (Table S1, Supporting Information) to be compared with each other, and with normal control (NC) and TGFβ‐treated microHOs (Figure 1D–F). For example, consistent with our prior results,^[^ ^12^ ^]^ TGFβ receptor (TGFBR1, SB431542), GSK3β (CHIR‐99021), and p38 (SB202190) inhibitors blocked TGFβ‐induced fibrosis but did not cause significant cellular toxicity in microHOs (Figure 1G,H; Figure S2F, Supporting Information). A PPARγ inhibitor had a small effect on TGFβ‐induced fibrosis in microHOs (Figure S2G, Supporting Information). Their relative positions in the heatmap indicates that the TGFBR1, GSK3β or p38 inhibitor‐treated microHOs were more like NC microHOs, while PPARγ‐treated microHOs were more like TGFβ‐treated microHOs (Figure 1D).

### 2.2 AI‐Assisted Repurposing of Drugs Targeting Epigenomic Pathways for Treatment of Liver Fibrosis

AI co‐scientist was asked to generate experimentally testable hypotheses about the role of epigenomic changes in liver fibrosis. In response, it hypothesized that: i) “*histone modifications, particularly deacetylation, in the promoter regions of genes responsible for MyoF differentiation”;* ii) *dynamic changes in DNA methylation patterns around genes involved in the MyoF pathway could be crucial; and* iii) *“pharmacological inhibition should be used to test the importance of epigenomic alterations.”* It also suggested that the role of epigenomics in liver fibrosis could be assessed by testing the anti‐fibrotic effect of inhibitors targeting 3 epigenomic modifiers: histone deacetylases (HDACs), which alter gene expression by removing the acetyl moieties from histones to create a more compact chromatin;^[^ ^14^ ^]^ DNA Methyltransferase 1 (DNMT1), which produces heritable changes in DNA methylation patterns;^[^ ^15^ ^]^ and bromodomain 4 (BRD4), which promotes transcriptional elongation by binding to acetylated lysine residues on histones to promote cell cycle progression and organ development^[^ ^16^ ^]^ (Data File S1, Supporting Information). For comparison, based upon a literature review, one author (GP) selected two other epigenomic targets: EZH2 in the Polycomb Repressive Complex 2 (PRC2) because EZH2 inhibitors reduced liver fibrosis in two murine models;^[^ ^17^ ^]^ and SMARCA2/4 in the Switch/Sucrose‐Nonfermentable (SWI/SNF) complex because it interacts with transcription factors that are activated in fibrotic liver,^[^ ^18^, ^19^ ^]^ and it inhibits a (Hippo) pathway that regulates hepatic stellate cell activation.^[^ ^20^ ^]^ However, inhibitors of the scientist selected targets (PFI‐3 for SMARCA2/4^[^ ^21^ ^]^ and GSK126 for EZH2^[^ ^22^ ^]^) did not reduce fibrosis in microHOs (Figure S2H, Supporting Information); and their relative positions in the heatmap indicated that the microHOs treated with these drugs resembled TGFβ‐treated microHOs (Figure 1D,F).

The drugs used to test the AI co‐scientist selected epigenomic targets were: a selective DNA methyl transferase 1 (DNMT1) inhibitor (GSK3685032) that is less toxic and has more durable hypomethylating effects than prior DNMT inhibitors;^[^ ^23^ ^]^ an FDA‐approved anti‐cancer drug that is a pan‐histone deacetylase (HDAC) inhibitor (Vorinostat or SAHA);^[^ ^24^ ^]^ and three BRD4 inhibitors: JQ1 (an early BRD4 inhibitor),^[^ ^25^ ^]^ and two (I‐BET762 (GSK525762),^[^ ^26^ ^]^ ZEN‐3694^[^ ^27^ ^]^) with improved risk‐benefit profiles. The DNMT1 inhibitor did not reduce fibrosis in microHOs; and its cytotoxicity would preclude further clinical development (Figure S2H, Supporting Information). In contrast, fibrosis was blocked by all 3 BRD4 inhibitors and by the HDAC inhibitor at concentrations that did not induce cellular toxicity (Figure 1I,J; Figure S2D,E,I,J, Supporting Information). The drug effect heatmap shows the BRD4 and HDAC inhibitor effects clustered together (Figure 1D,F). Like the correlations calculated using the mean feature values, we also assess similarity using k‐mean clustering based on the imaging data matrix, and 8 optimal clusters were identified from analysis of 16 333 individual microHOs. The cluster distribution (Figure 1E) generated for 25 drug treatments (Figure S2A–C, Supporting Information) show that TGFRB1i and p38i treated HOs are like NC, while the BRD4i, HDACi and GSK3βi generate unique clusters (Figure 1F).

**Figure 2:**
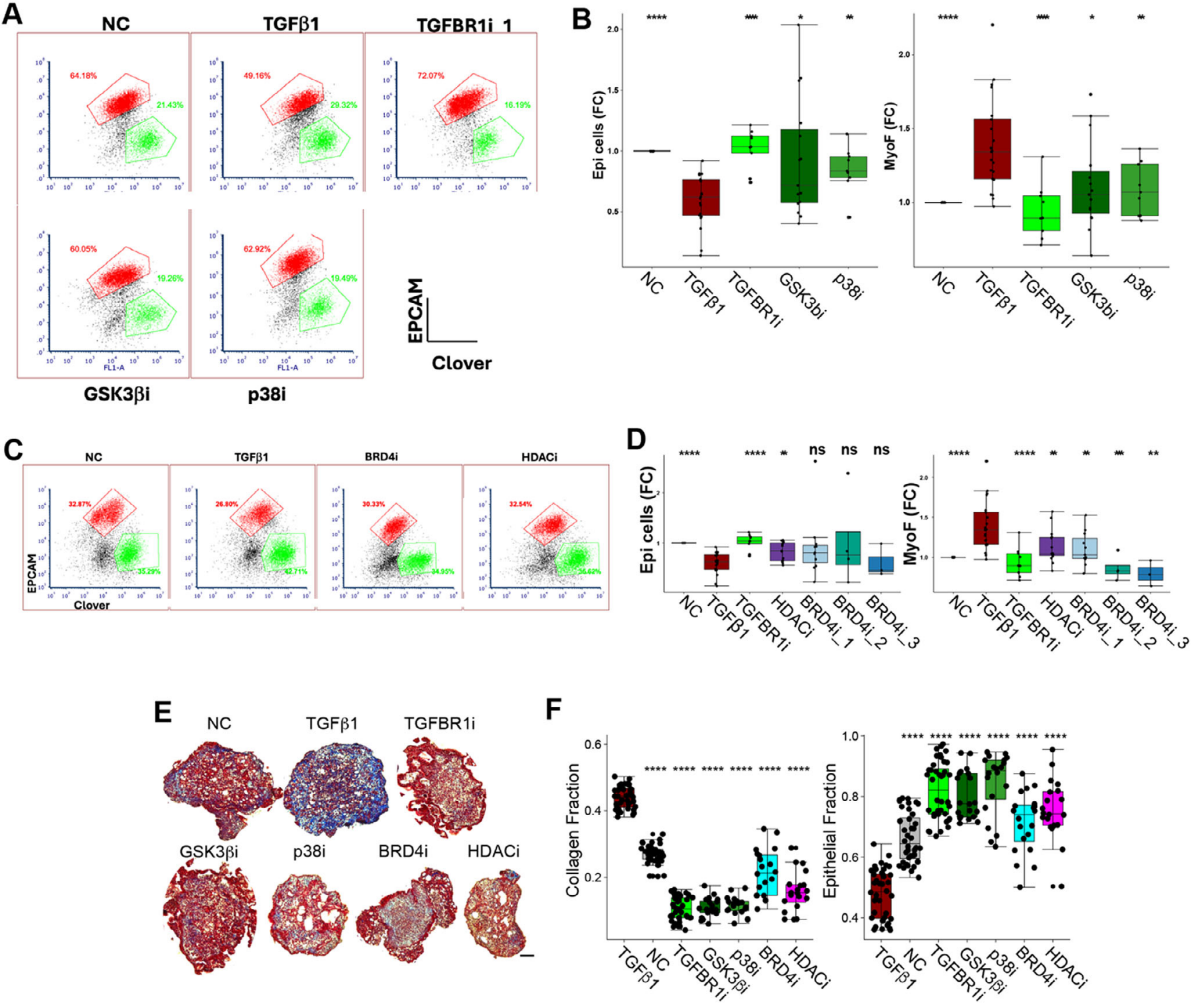
Inhibitors block TGFβ‐induced fibrosis and promote liver regeneration in microHOs. A,C) Representative FCM results obtained from day 20 microHOs treated with normal media (NC), TGFβ1, or TGFβ1 and drug (GSK3βi 3 µm, TGBR1i 10 µm, p38i 10 µm, BRD4i 2 µm, and HDACi 1 µm.). The x‐ and y‐axes shows the COL1A1 Clover fluorescence the cells stained with an anti‐EPCAM antibody, respectively. B,D) These plots show the change in epithelial cells (Epi) and Clover^+^ cells (MyoF) in microHOs caused by the indicated treatments. Each FCM measurement is made on at least 10 individually prepared batches of microHOs. To calculate the fold change (FC), the percentage of cells in each batch was normalized relative to that in NC microHOs. SB431542 is the TGFBR1 inhibitor; Vorinostat (SAHA) is the HDAC inhibitor; and ZEN‐3694, IBET762 and JQ‐1 are the BRD4 inhibitors 1 to 3 (whose concentrations are as above). ns, not significant; *p‐value < 0.05; **p‐value < 0.001; ***p‐value < 0.001; and ****p‐value < 0.0001. E) Images of Trichrome‐stained TGFβ1‐treated microHOs on day 20 show a marked increase in collagen‐rich connective tissue (blue‐stained regions) relative to NC microHOs, which only had a thin layer of connective tissue. The TGFβ1‐induced increased in collagen was markedly inhibited by co‐addition of TGFBR1, HDAC, or BRD4 inhibitors (concentrations as indicated above). Moreover, the number of epithelial cells (dark red regions) was decreased by TGFβ1, and co‐addition of TGFBR1, HDAC, or BRD4 inhibitors prevented the decrease in epithelial cells. Scale bar: 100 µm. F) Box plots show the area within day 20 microHOs that received the indicated treatment (n > 10 per group) occupied by collagen (collagen fraction) or parenchymal cells (epithelial fraction). ns, not significant; *p‐value < 0.05; **p‐value < 0.001; ***p‐value < 0.001; and ****p‐value < 0.0001.

FCM results confirmed that the 5 drugs (TGFBR1, p38, GSK3β, HDAC, and BRD4 inhibitors) that decreased fibrosis (i.e., clover expression) also reduced the number of collagen producing cells in TGFβ‐treated microHOs. FCM results also revealed that the TGFBR1, p38, GSK3β, and HDAC inhibitors increased the number of liver parenchymal (EPCAM^+^) cells in TGFβ‐treated microHOs (**Figure** **2**A–D); while the BRD4i induced‐increase in epithelial cells did not reach statistical significance. However, trichrome staining confirmed that these 5 inhibitors decreased the amount of ECM (*p* < 0.001) and increased the amount of liver parenchymal cells (*p* < 0.001) in TGFβ‐treated microHOs (Figure 2E,F). Hence, these drugs not only reduced fibrosis but promoted liver parenchymal cell regeneration in the presence of a pro‐fibrotic stimulus.

**Figure 3:**
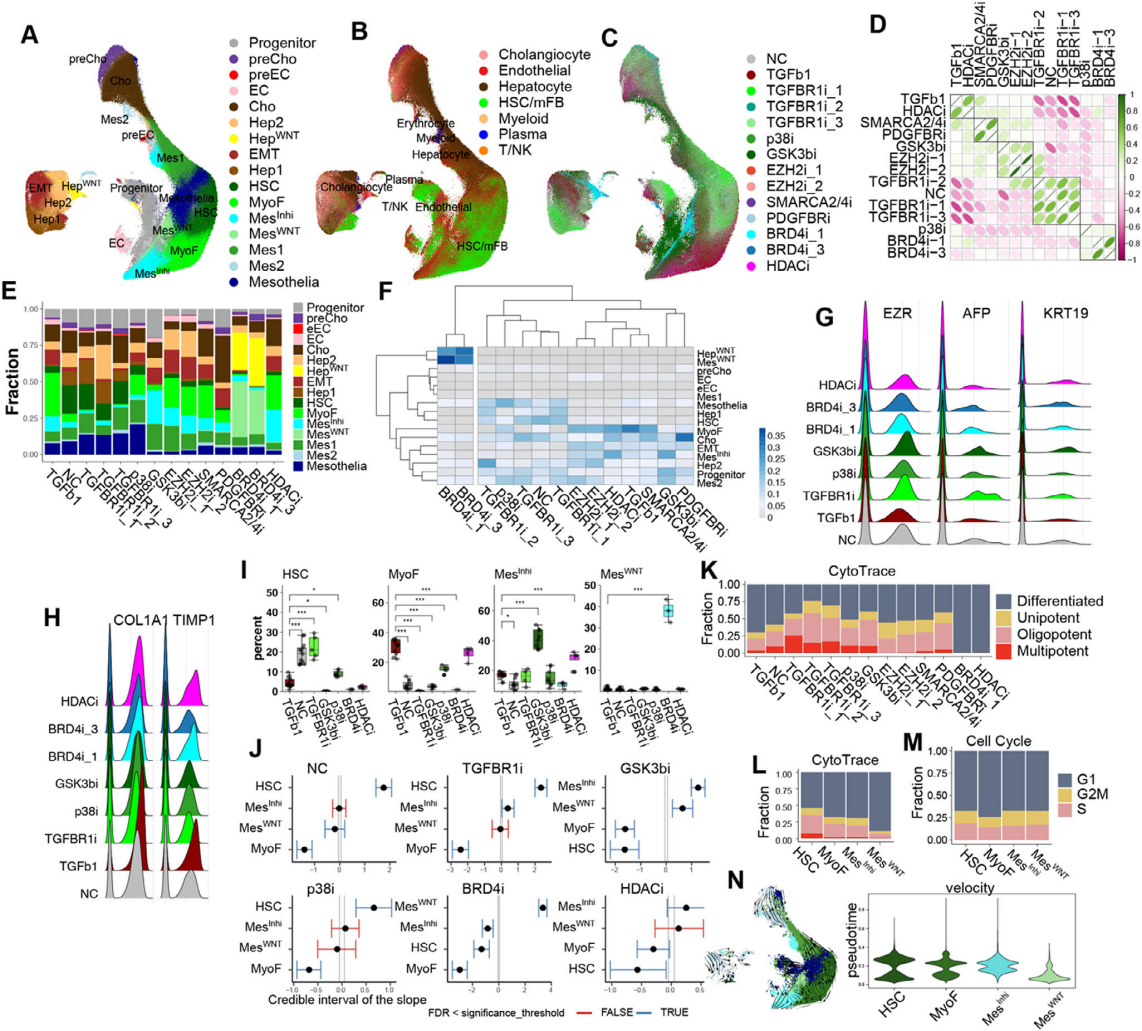
TGFβ1 and drug‐induced cellular and transcriptomic changes in microHOs. A–C) UMAP plots are labeled with the 16 cell clusters identified by canonical markers (A) and Azimuth auto labelled 7 clusters (B) or by the microHOs receiving 14 different treatments (C) in microHOs. scRNA‐seq data was generated from 613826 cells obtained from microHOs receiving the indicated 14 treatments. D) A heatmap showing the relationships between the mean transcriptomes of normal control (NC), TGFβ, and TGFβ plus drug‐treated microHOs. The Pearson correlation coefficient for each transcriptomic comparison was calculated using the scRNA‐seq data described in (C). E) scRNA‐seq data obtained from NC, TGFβ, and TGFβ plus drug treated microHOs were analyzed to identify the percentage of cells in each of the 16 cell clusters in each type of microHO. The percentage of each indicated cell type represents the average obtained from 17 experimental batches. F) A heatmap shows the hierarchical clustering of the percentage of each of the 16 cell clusters in NC, TGFβ1 or TGFβ1 plus drug‐treated microHOs. G) RidgePlots show the decreased epithelial population (*EZR^+^, AFP^+^*, and *KRT19^+^*) in TGFβ‐treated microHOs, but this population is restored in microHOs treated with the indicated drugs. H) RidgePlots show the increased mesenchymal population (*COL1A1^+^, TIMP1^+^*) in TGFβ‐treated microHOs, but this increase is inhibited in drug treated microHO. I) Graphs comparing the changes in the percentage of 4 mesenchymal cell types in the microHOs shown in (A). Each group is the average of microHOs prepared from 5 to 10 separate experiments, and the bars show the mean±SEM. ANOVA test p‐values for the for the indicated comparisons are *, < 0.05; **, < 0.01; or ***, < 0.001. J) A plot of the estimates of the differential cell composition (determined by sccomp analysis) for four mesenchymal cell types in NC or TGFβ+drug treated microHOs from (A). The percentages of each cell type were compared with those in TGFβ treated microHOs. The blue bars indicate that there was a significant difference versus TGFβ‐treated microHOs. K) Cell potency categories are plotted as a percentage bar plot that was generated by “Cytotrace” analysis of scRNA‐seq data generated from NC, TGFβ or TGFβ plus drug‐treated microHOs. L) The “Cytotrace” cell potency categories are plotted for four mesenchymal clusters. M) The “cell cycle” categories are plotted for the 4 mesenchymal clusters. N) The velocity vector field is displayed as streamlines of mesenchymal clusters that are projected onto the UMAP embedding (left). The predicted “velocity_pseudotime” for the 4 mesenchymal clusters in microHOs (right).

Molecular analysis of anti‐fibrotic drug effects. Since AI co‐scientist suggested that we use “single cell RNA sequencing (scRNA‐seq) to assess global transcriptional changes associated with fibrosis and drug effects,” scRNA‐seq data was generated from 613826 cells obtained from NC microHOs, TGFβ‐ and TGFβ plus drug treated microHOs. (The number of cells evaluated for each drug, cell type and the number of experimental batches are listed in Data File S2A–C, Supporting Information). UMAP plots show the sixteen cell clusters (7 epithelial, 7 mesenchymal, and 2 endothelial clusters) identified in microHOs, which were annotated based upon transcriptomic comparisons with reference datasets containing fetal, adult normal, and diseased human liver samples; and based upon canonical marker mRNA expression (**Figure** **3**A–C; Figure S3A–F, Supporting Information). A detailed characterization of the differentially expressed genes (DEG) and active pathways in the clusters are described in Note S2 (Supporting Information); and the properties of the key clusters are summarized here. Of importance, microHOs have clusters resembling the MyoF and hepatic stellate cells (HSC) found in human liver. One mesenchymal cluster (Mes^Inh^) had DEGs with anti‐fibrotic activity (ECM1,^[^ ^28^ ^]^ PI16,^[^ ^29^ ^]^ CD47,^[^ ^30^ ^]^ and CD109^[^ ^31^ ^]^)), while another (Mes^WNT^) had activated WNT signaling. A Progenitor cluster resembled the progenitor cells in fetal liver; the Hep^WNT^ cluster resembled a differentiated hepatocyte and had an activated WNT pathway; and Hep1 and Hep2 cells resembled hepatocytes and hepatocyte precursors, respectively. The EMT cells had DEG characteristic of the epithelial to mesenchyme transition process, and resembled EPCAM^+^ fetal and adult liver cells. The preCho and Cho cells had similarities with bipotent progenitor cells or cholangiocytes, respectively.

The scRNA‐seq data was examined to investigate how five drugs (GSK3βi: CHIR‐99021, p38i: SB202190, TGFBR1i: SB431542, BRD4i: ZEN‐3694, and HDACi: SAHA) exerted their anti‐fibrotic effects. Analysis of drug‐induced changes in cell type abundance revealed that the TGFBR1i and p38i‐treated microHOs resembled NC microHOs, which is consistent with an effect on their targeted pathways (Figure 3D–F). The TGFBR1i blocks TGFβ‐induced intracellular signaling, which also activates the pro‐fibrotic p38 Mitogen Activated Protein Kinase (MAPK) signaling pathway.^[^ ^32^ ^]^ We previously found that the MAPK pathway was activated in MyoF in microHOs and in cirrhotic human liver,^[^ ^12^ ^]^ and these results confirm that its activation is essential for TGFβ‐induced liver fibrosis. All five drugs decreased the cell populations expressing mesenchymal markers (*COL1A1, TIMP1)* and increased those expressing epithelial markers (*EZR*, *AFP*, *KRT19, CDH1, EPCAM*, *and CYP1B1)* (Figure 3G,H; Figure S3H, Supporting Information), while the HDACi and BRD4i had distinct effects on the cellular composition (Figure 3D,F). In contrast, the PDGFRBi, SMARCA2/4i and EZH2i did not decrease the cell populations expressing mesenchymal markers (Figure S3G, Supporting Information). Since seventeen different experimental batches of microHOs were analyzed, drug‐induced changes in cluster abundance were analyzed by ANOVA testing and using a mixed effect model (“sscomp”)^[^ ^33^ ^]^ (Figure 3I,J). While all 5 drugs prevented the TGFβ induced increase in MyoF, only the TGFBR1i prevented the TGFβ‐induced decrease in HSC. Of relevance to their anti‐fibrotic effect, Mes^Inh^ cells were induced by GSK3βi and HDACi; and the BRD4i dramatically increased Mes^WNT^ abundance.

Drug‐induced effects on the cellular differentiation state of microHO clusters was determined by analysis of the transcriptomic data using the “Cytotrace” and “Velocity” programs. While TGFβ significantly decreased the pool of progenitor cells, the GSK3βi and TGFBR1i inhibitors prevented the loss of the stem cell pool (Figure 3K; Figure S8A,B,F–H, Supporting Information), but the GSK3βi acts via a different mechanism than the TGFRB1 or p38 inhibitors. Although the GSK3βi does not block TGFβ‐induced intracellular signaling, it alters the TGFβ‐induced changes in cellular differentiation. The TGFβ, Wnt/β‐catenin and p38 pathways have multiple interaction points in liver^[^ ^34^ ^]^ and during development,^[^ ^35^ ^]^ which might explain how a GSK3β inhibitor alters TGFβ‐induced cellular differentiation. In contrast, the progenitor pool was dramatically decreased in BRD4i or HDACi treated microHOs, which indicates that those epigenomic drugs strongly induce cellular differentiation (Figure 3L,N). However, the drug‐induced effects on cellular differentiation occurred without a change in cell cycle (Figure 3M; Figure S8C–E, Supporting Information).

**Figure 4:**
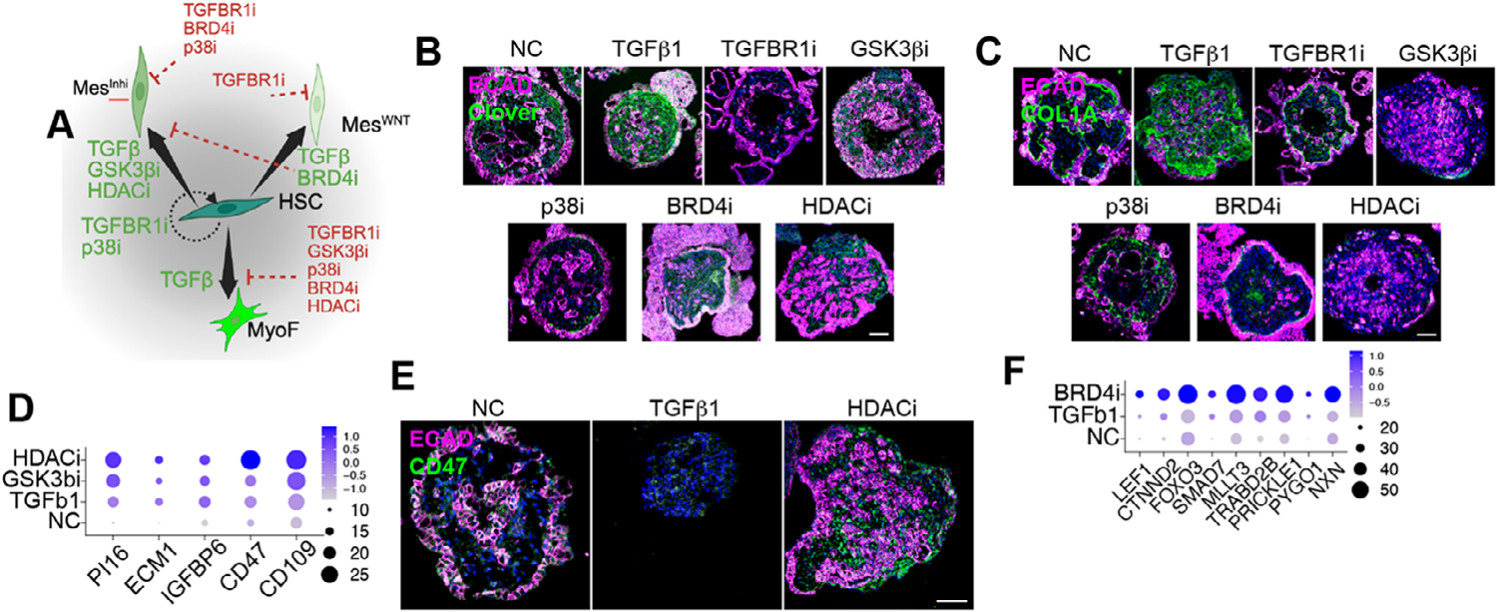
Anti‐fibrotic drug induced effects on mesenchymal cell type in microHOs. A) A diagram summarizing the effects of TGFβ and drugs on mesenchymal cells in microHOs. HSC in normal HOs are stimulated by TGFβ to differentiate into MyoF, which is reduced by co‐administration of the indicated drugs. GSK3βi and HDACi alter HSC differentiation to generate an anti‐fibrotic mesenchymal cell type (Mes^Inhi^), while BRD4i promotes the generation of Mes^WNT^ cells. B,C) Immunostaining shows Clover^+^ cell population and COL1A deposition in HOs with indicated treatments. D) This dot plot shows average level of expression and the percent of cells expressing Mes^Inh^ marker mRNAs (*Pl16, ECM1, IGFBP6 and CD109*) in day 21 NC, TGFβ1‐treated or TGFβ1 + drug (HDACi: 1 µm or GSK3βi: 3 µm) treated microHOs. indicated treatments. Dot color indicates the relative level of expression and dot size indicates the percent of cells expressing the indicated mRNA according to the key shown on the right. E) Immunostaining shows ECAD and Mes^Inh^ marker CD47 protein expression in NC, TGFβ‐treated or TGFβ + HDACi‐treated day 21 microHOs. CD47 expression is increased in HDACi‐treated microHOs. F) This dot plot shows average level of expression and the percent of cells expressing Mes^WNT^ marker proteins in day 21 NC, TGFβ1‐treated or TGFβ1 + BRD4i‐treated (2 µm) microHOs. Dot color indicates the relative level of expression and dot size indicates the percent of cells expressing the indicated mRNAs according to the key shown on the right. The BRD4i increases Mes^WNT^ marker mRNA expression.

In summary, TGFβ causes HSC to differentiate into MyoF in microHOs, and this can be prevented by all five anti‐fibrotic drugs (**Figure** **4**A). The GSK3βi and HDACi generate inhibitory mesenchymal cells (Mes^Inh^), and the BRD4i induces the formation of another inhibitory mesenchymal cell type (Mes^WNT^), which explains their anti‐fibrotic effect. While the BRD4i and HDACi had different effects on mesenchymal cells, both promote progenitor cell differentiation into more specialized cell types. Immunostaining confirmed that TGFβ‐induced fibrosis (increased Clover^+^ and Collagen deposition) is suppressed by the 5 anti‐fibrotic drugs (Figure 4B,C). scRNA‐seq data confirms that Mes^Inh^ mRNA (*PI16*, *ECAM1*, *CD109*, and *CD47)* expression is increased in HDACi‐treated microHOs (Figure 4D). Immunostaining also shows that increased Mes^Inh^ protein (CD47) expression in HDACi treated microHOs (Figure 4E). Mes^WNT^ marker mRNA (*CTNND1*, *FOXO3*, and *PRICKLE1)* expression is increased in BRD4i treated microHOs (Figure 4F).

**Figure 5:**
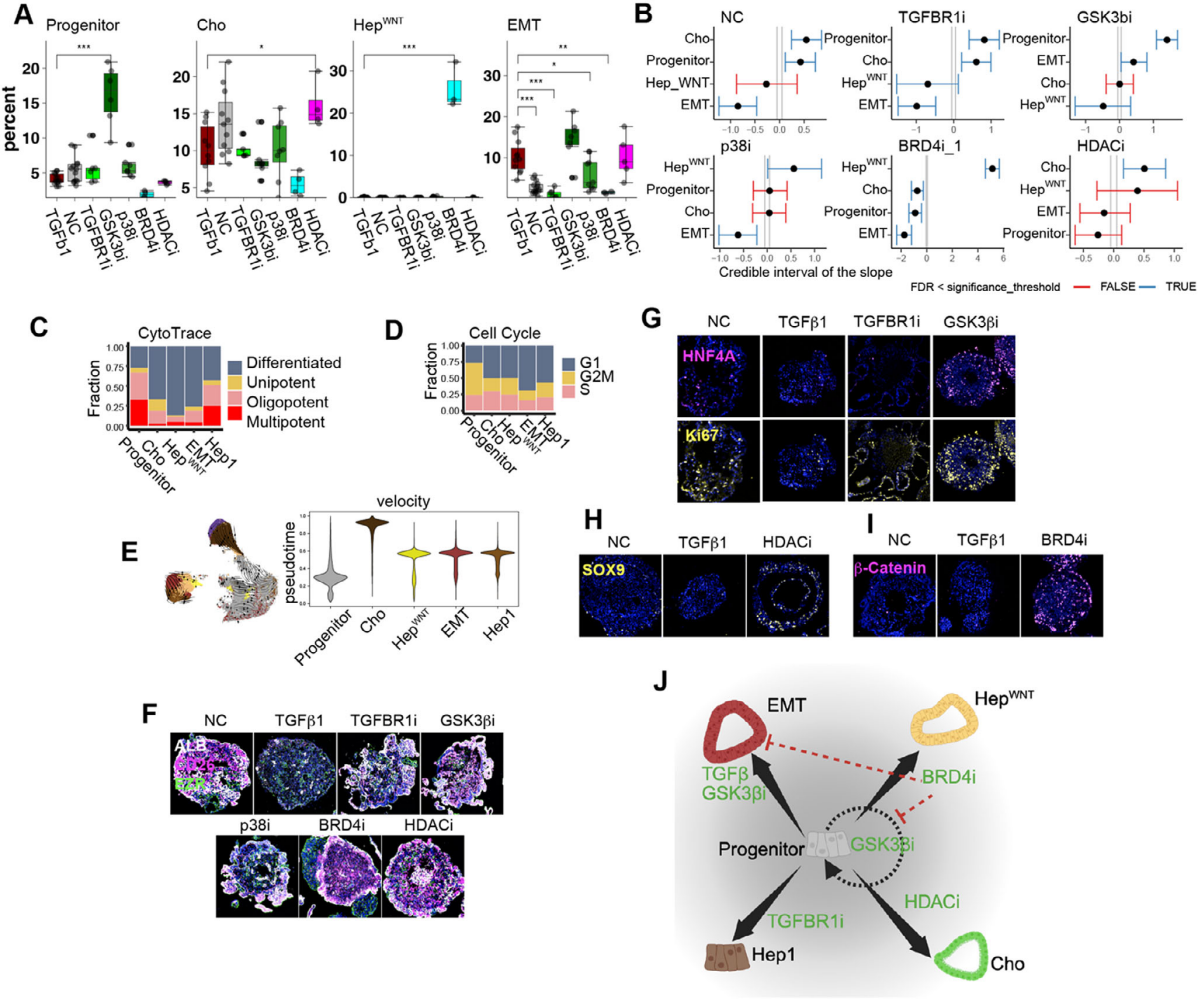
Anti‐fibrotic drug effects on liver epithelial cells in microHOs. A) Graphs comparing the changes in the percentage of the 4 epithelial cell types in microHOs. Each group is the average of microHOs prepared from 5 to 10 separate experiments, and the bars show the mean±SEM. ANOVA test p‐values for the for the indicated comparisons are *, < 0.05; **, < 0.01; or ***, < 0.001. B) A plot of the estimates of the differential cell composition (determined by sccomp analysis) for four types of epithelial cells in NC or TGFβ+drug treated microHOs prepared as in (3A). The percentages of each cell type were compared with those in TGFβ treated microHOs. The blue bars indicate that there was a significant difference versus TGFβ‐treated microHOs. C,D) The “Cytotrace” cell potency categories (C) and “cell cycle” categories (D) are plotted for four of the epithelial clusters in microHOs. These plots show that the drugs alter the differentiation status of epithelial cells, but this effect does not involve a cell cycle changes. E) The velocity vector field is displayed as streamlines of 4 epithelial clusters that are projected onto the UMAP embedding (left). The predicted “velocity_pseudotime” for the 4 epithelial clusters in microHOs is shown on the right. These results show the transitional cell type velocity among the different epithelial clusters, which progress from the Progenitor to the Cho cluster. F) Immunostaining shows the liver parenchymal population expressing epithelial markers (ALB, CD26 and EZR) in day 21 NC, TGFβ‐treated, or TGFβ + drug‐treated (TGFBR1i, GSK3βi, p38i, BRD4i, HDACi) microHOs. G‐I) Immunostaining of day 21 microHOs shows that: TGFBR1i or GSK3βi treatment increases the Ki67^+^ cell population (G), HDACi increases the SOX9^+^ cell population (H); and BRD4i increases the number of cells with active (non‐phosphorylated) β‐Catenin^+^ (I). J) This illustration shows that hepatic progenitor cells will differentiate into normal hepatocytes (Hep1) when TGFBR1i is co‐administered with TGFβ. However, TGFβ1 and GSK3β treatment will induce the production of an EMT like population; while  co‐administration of a BRD4i or HDACi can convert them into Hep^WNT^ and cholangiocytes, respectively.

TGFβ and the inhibitors also had significant effects on the 7 parenchymal epithelial clusters in microHOs. Only the TGFBR1i could revert the TGFβ‐induced loss of hepatocytes. While TGFβ significantly decreased the abundance of the preCho and Hep2 clusters, their abundance was not altered by the GSK3β, p38 or TGFBR1 inhibitors. The GSK3βi increased Progenitor cell abundance (**Figure** **5**A,B). While the TGFBR1i and p38i suppressed the TGFβ‐induced increase in EMT cells, the GSK3β inhibitor expanded this cluster. The BRD4i and HDACi increased Hep^WNT^ and Cho cell abundance, respectively, which were the most differentiated epithelial clusters (Figure 5C,E). The HDACi also increased EMT cell abundance. While TGFβ significantly decreased the progenitor cell pool, the BRD4i and HDACi increased the level of epithelial cell differentiation, and this effect was not due to cell cycle changes (Figure 5D; Figure S8C–H, Supporting Information). These findings indicate that while all 5 inhibitors had different mechanisms for promoting epithelial cell differentiation, they all promoted liver regeneration. The drug‐induced increase in parenchymal cells was confirmed by immunostaining of parenchymal markers (ALB, CD26, EZR, CK8 and Par1) in microHOs (Figures 4, 5; Figure S8I,J, Supporting Information). Analysis of immunostained microHOs also revealed that: the GSK3βi increased the Ki67^+^/HNF4A^+^ population (Figure 5G); HDACi increased SOX9^+^ cells (Figure 5H); and BRD4i increased the amount nuclear trans‐located β‐Catenin (Figure 5I). These findings indicate that: i) WNT signaling is essential for maintaining the liver progenitor pool, which can be expanded through an effect of GSK3βi on the Progenitor and EMT cluster; ii) BRD4i can expand the Hep^WNT^ cluster via an effect on the RSPO5/FZD5 pathway; and that the HDACi promotes cholangiocyte differentiation (Figure 5J). Thus, all 5 inhibitors could promote liver regeneration, but they utilized different mechanisms for doing this.

### 2.3 Analysis of Chromatin Structural Changes

**Figure 6:**
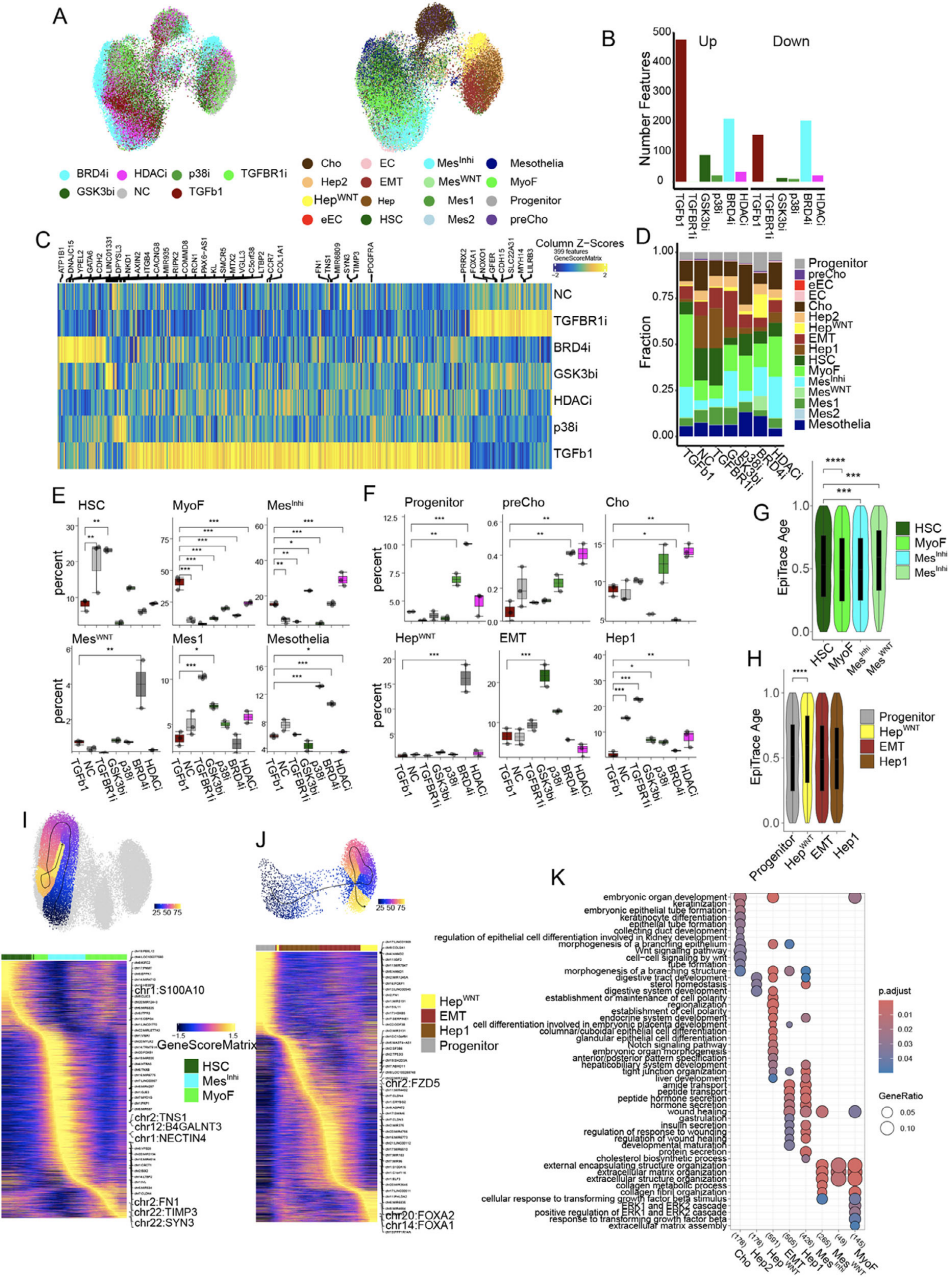
TGFβ1 and drug‐induced epigenetic changes in microHOs. A) Combined sample UMAP plots of the snATAC‐seq data generated from microHOs that received the indicated treatments (Left); or with labeling of the 16 cell clusters, which were determined by transfer of the scRNA‐seq data coordinates (Right). The data for each group was generated from three different experimental batches of samples. B) Bars plot illustrates the number of differential marker genes identified by comparing the gene scores between each specified treatment and normal control (NC) microHOs. These comparisons are made to ensure that the TSS (Transcription Start Site) enrichment ratio and the log_10_‐transformed number of fragments are similar between the treatment and NC groups. The “up” or “down” bars indicate whether the gene scores in the treatment group were higher or lower than in the NC group. The Wilcoxon test was used to analyze the differences in the gene scores between the treatment and NC groups for each gene. This statistical test identifies genes with significant differences in their gene scores, which highlights those that are differentially affected by treatment with TGFβ or TGFβ and drug. C) A heatmap shows the z‐score scaled “differential gene score” calculated from snATAC‐seq data for 248 features, which are organized by the indicated treatment. D) The average percentage of each cell type present in NC, TGFβ‐ or TGFβ+drug‐treated microHO. E,F) These graphs show the change in the percentage of six epithelial (E) or six mesenchymal (F) cell types in microHOs receiving the indicated treatment compared to TGFβ. The bars show the mean ± SEM. ANOVA test p‐values for the for the indicated comparisons are *, < 0.05; **, < 0.01; or ***, < 0.001. G,H) Single cell age estimates generated by EpiTrace using the scATAC‐seq data are plotted for the mesenchymal (G) and epithelial (H) clusters. This data shows that the drugs have different effects on the epigenetic aging status for the mesenchymal and epithelial clusters. ***p‐value < 0.001; and ****p‐value < 0.0001. I,J) *Upper panels*: Cell differentiation trajectories were determined by analysis of scATAC‐seq data for mesenchymal (I) and epithelial (J) clusters. *Lower panels*: pseudo‐time heatmaps showing the differential gene scores for the indicated marker genes in day 21 microHOs. These results indicate that the HSC‐Mes^Inh^‐MyoF and the Progenitor‐Hep1‐EMT‐Hep^WNT^ axes proceed along separate developmental paths. K) Over representation analyses (ORA) showing mRNA marker gene expression in day 21 microHOs from analysis of scATAC‐seq data for 4 mesenchymal and 4 epithelial clusters. The DEGs for each cell type were annotated using the GO‐BF databases. The key on the right shows the gene ratio, which refers to the proportion of genes from input gene list that are associated with a reference term or category.

Since AI co‐scientist also recommended that “the Assay for Transposase‐Accessible Chromatin sequencing (ATAC‐seq) be used to map the TGFβ‐ and drug‐induced effects on accessible chromatin regions,” snATAC‐seq was used to analyze NC, TGFβ‐treated, and TGFβ plus drug‐treated microHOs. Quality control metrics (length and number of fragments, and transcription start site (TSS) enrichment) indicated that the ATAC‐seq data was of high quality (Figure S9A–C, Supporting Information), and snATAC‐seq UMAP plots corresponded with the scRNA‐seq UMAP plots (**Figure** **6**A). Since differences in chromatin structure are an indicator of differential gene expression, chromatin structural changes near TSS were identified by calculating differential gene scores when different treatment groups or cell clusters were compared. There were 3527 marker genes with differential gene scores when snATAC‐seq results for the 16 cell clusters were compared. Of interest, hepatocytes had the highest number of differential gene scores (Figure S9D,E, Supporting Information). The dramatically different effects of TGFβ‐ or TGFβ+drug treatment is seen in a heatmap that profiles the differential gene scores (n = 399) for each condition. To examine the effect of each drug on TGFβ‐induced chromatin structural changes, the differential gene scores for TGFβ− or TGFβ +drug‐treated microHOs were individually compared with NC microHOs. From these comparisons, 636 genes had differential gene scores (478 increased, 158 decreased) in the TGFβ‐treated versus NC microHOs, and the TGFBR1 inhibitor blocked all TGFβ‐induced chromatin structural changes (Figure 6B,C). The p38i and GSK3bi reduced the number of differential gene scores by 94.3% or 84% (versus TGFβ), respectively. The BRD4i had a lesser effect (43% reduction) and the HDACi caused a 91% reduction in TGFβ‐induced chromatin structural changes. Most importantly, the TGFβ‐ and drug‐induced chromatin structural changes in the cell types determined by analysis of snATAC‐Seq data reflected those measured using scRNA‐seq data (Figure 6D–F). Specifically, TGFβ induced a significant increase in the percentage of MyoFs, which was blocked by all 5 tested drugs. The drug‐induced changes in cellular composition determined by analysis of scRNA‐seq data were confirmed by analysis of the snATAC‐seq data. Only the TGFBR1i could rescue the loss of HSC; the GSK3β and HDAC inhibitors diverted mesenchymal cells to become Mes^Inh^; and BRD4i diverted mesenchymal cells to Mes^WNT^. TGFβ also caused a significant decrease in the percentage of hepatocytes, which was countered by the TGFBR1i, GSK3βi and HDACi. The percentage of preCho cells was increased by the BRD4i and HDACi; and the BRD4i increased the abundance of the Hep^WNT^ cluster.

To understand the mechanisms underlying the drug‐induced changes, “EpiTrace” was used to reconstruct cell developmental trajectories from analysis of scATAC‐seq data.^[^ ^36^ ^]^ The Mes^Inh^, Mes^WNT^, and Hep^WNT^ cells have distinct EpiTrace‐determined Age scores (Figure 6G,H). We also calculated approximate differentiation pseudotime and cellular trajectories (Figure 6I,J) for the mesenchymal and epithelial clusters, respectively. The results indicated the HSC‐Mes^Inh^‐MyoF and the Progenitor‐Hep1‐EMT‐Hep^WNT^ axes proceed along separate developmental paths. Moreover, the differential gene scores obtained for those clusters are like those obtained using the scRNA‐seq data (Figure 6K). For example, genes effecting epithelial cell differentiation and hepatobiliary system development are enriched in the Hep^WNT^ cluster, while genes involved in ECM assembly and the TGFβ response are enriched in MyoF. Hence, analysis of chromatin structural changes confirmed that multiple mechanisms underlie the anti‐fibrotic and liver regenerative effects of these 5 drugs.

## 3 Discussion

This study provides a first demonstration that a compound, multi‐agent system, which was designed to mirror the reasoning process underlying scientific discovery, can assist in re‐purposing drugs for treating a disease with limited therapeutic options. Two of three types of epigenomic modifier drugs recommended by AI co‐scientist exhibited significant anti‐fibrotic activity, and both had features that make them excellent candidates for treating liver fibrosis. We recently demonstrated that gene‐phenotype analyses performed by a large language model (LLM) could lead to novel genetic discoveries in mice and genetic diagnoses in humans.^[^ ^37^ ^]^ The results presented here demonstrate that a next generation LLM system (AI co‐scientist) can analyze the vast amounts of scientific literature covering a specified area, identify hidden connections, and generate novel hypotheses that could produce new therapeutic approaches. Remarkably, AI co‐scientist also guided the research steps used for testing the hypothesis. Due to the inherent complexity of biomedical research, AI‐generated hypotheses require rigorous experimental validation by scientists and the hypotheses must be carefully reviewed by scientists. The purpose of these AI tools is to augment (and not replace) human scientific reasoning; their use will increase the ability of researchers to generate discoveries, while allowing them to retain intellectual oversight of the discovery process. Nevertheless, this work represents a significant milestone for AI, it indicates how AI‐enabled science could significantly accelerate discoveries that could advance many biomedical fields.

Drug repurposing is now an important area for biomedical research, since it can produce new treatments for diseases.^[^ ^38^ ^]^ However, repurposing a drug for even one disease requires that a scientist must have a comprehensive understanding of the pathobiology of that disease along with knowledge about the therapeutic target and possible effects of each drug (among the many drugs that have been approved for treatment of other diseases) on the disease‐causing pathway(s). Computational methods using knowledge graphs or graph convolutional networks have shown promise for drug repurposing;^[^ ^39^ ^]^ and a specialized graph‐based computational approach^[^ ^40^ ^]^ was recently developed for repurposing of drugs for novel diseases. However, these graph‐based approaches are limited by the quality of the underlying graph, they lack scalability and cannot fully explain the drug‐disease connections; nor can they design the methods for experimental testing of the recommended drugs. In contrast, AI co‐scientist is a scalable, state‐of‐the‐art LLM, which uses a multi‐agent system that can make complex connections between drug effects and disease pathobiology for drug re‐purposing. Moreover, it can also provide the reasons underlying the drug‐disease connections that it makes and can recommend experimental methods for testing the effect of its recommended drugs on disease biology.

Our results demonstrate that the microHO platform provides a robust, high‐throughput platform for evaluating the anti‐fibrotic and cytotoxic effects of drug candidates in a developing hepatic organoid. Moreover, the FCM, scRNA‐seq and ATAC‐Seq data provide key information about drug‐induced effects on parenchymal cell regeneration, cell differentiation state, and cell composition in a fibrotic liver‐like environment. The TGFBR1, GSK3β, p38 and HDAC inhibitors reduce fibrosis and increased the number of hepatic parenchymal cells after TGFβ exposure. Transcriptomic analyses revealed that their anti‐fibrotic effects are mediated by different mechanisms. TGFBR1 and p38 inhibitors blocked TGFβ‐induced intracellular signaling; while GSK3β, BRD4 and HDAC inhibitors altered TGFβ‐induced mesenchymal cell differentiation. Vorinostat (Mes5), GSK3β (Mes5) and BRD4 (Mes6) inhibitors caused a marked increase in mesenchymal cell populations that were not present in NC or TGFβ‐treated microHOs. While BRD4 inhibitors are still in clinical trials; Vorinostat^[^ ^24^ ^]^ is an FDA approved anti‐cancer drug, which did not cause toxicity in microHOs nor did it cause hepatitis or clinically apparent liver injury in treated subjects.^[^ ^41^ ^]^ Vorinostat is being considered as a treatment for other types of cancers,^[^ ^42^ ^]^ but it had not previously been considered for treatment of liver fibrosis. A very limited amount of prior experimental data supported its potential efficacy in liver fibrosis: it suppressed hepatic stellate cell activation in vitro,^[^ ^43^ ^]^ and reduced liver injury^[^ ^44^ ^]^ and fibrosis^[^ ^45^ ^]^ in rodent models. Since microHOs do not have immune cells, the observed anti‐fibrotic effects result from decreased production of activated myofibroblasts. Since immune cells in the liver play an important role in the pathogenesis of many liver diseases, it will be important to develop methodology to incorporate Kupfer cells into microHOs.^[^ ^11b^ ^]^ Because epigenomic modifications effect gene expression and cellular differentiation, they play a role in the pathogenesis of multiple human diseases^[^ ^46^ ^]^ (including liver fibrosis^[^ ^47^ ^]^), and HDAC‐mediated effects on histones play a particularly important pathogenetic role.^[^ ^48^ ^]^ Vorinostat and BRD4i caused substantial reductions in TGFβ‐induced chromatin structure changes, which indicates that this is likely to be a major contributor to their anti‐fibrotic effect. It was also noteworthy that Vorinostat and BRD4 inhibitors potently induced cellular differentiation in microHOs. Anti‐cancer drugs, including Vorinostat and BRD4 inhibitors, have been used as cellular differentiating agents for treatment of several types of cancer;^[^ ^49^ ^]^ and Vorinostat can induce mesenchymal cell differentiation.^[^ ^50^ ^]^ Our findings indicate that epigenomic modifying drugs should be given serious consideration as treatments of liver fibrosis and could form part of a new generation of anti‐fibrotic agents that also promote liver parenchymal cell regeneration. Moreover, the COL1A1‐P2A‐Clover iPSC line can be used for modeling fibrosis that occurs in other organs, many of which can also be modeled using human organoids.

## Conflict of Interest

The Stanford University Medical School and ARC Institute authors have no competing interests. APaw, JG, AD, AM, Apal, KK, WH, VN, AK, KR, YX, and TT are employees of Alphabet and may own stock as part of their standard compensation package.

## Author Contributions

G.P. and Y.G. formulated the project. Y.G., T.T., and G.P. wrote the paper with input from all authors. Y.G., Z.C., L.C., J.I., Z.F., J.L., and A.A.G.B. generated experimental data. Y.G., L.C., APaw, J.G., A.D., A.M., A. Pal, L.R., K.K., W.W., V.N., A.K., K.R., Y.X., T.T., and G.P. analyzed the data. All authors have read and approved of the manuscript.

## Supporting information



Supporting Information

Supplemental Tables

Supporting Information

## Data Availability

All raw and processed single cell RNA‐seq data were deposited in the Gene Expression Omnibus (GEO) and are available under accession numbers (GSE228214 and GSE306096). Processed scRNA‐seq and snATAC‐seq data are also available at https://doi.org/10.5281/zenodo.14996331. We do not open‐source the model code and weights due to the safety implications of unmonitored use of AI co‐scientist. In the interest of responsible innovation, we will be working with research partners, regulators, and providers to validate and explore safe onward uses of AI co‐scientist, and we expect to make them available via Google Cloud APIs. For reproducibility, we have documented technical deep learning methods while keeping the paper accessible to a clinical and general scientific audience. Our work builds upon Google Gemini whose technical details have been described extensively in a technical report.^[^ ^51^ ^]^
