# Supplementary material for: AI‐Assisted Drug Re‐Purposing for Human Liver Fibrosis
Source: Adv Sci (Weinh). 2025 Sep 14;12(44):e08751. doi: 10.1002/advs.202508751 (PMC12667525; doi:10.1002/advs.202508751)
Supplement: Supplementary file 1 — Supporting Information [file ADVS-12-e08751-s001.docx]

**Supplemental Information**

**AI-assisted Drug Re-purposing for Human Liver Fibrosis**

*Supplemental note 1: AI co-scientist description.* AI co-scientist [1] is a multi-agent system built on Gemini 2.0 that is designed to mimic the scientific method and accelerate scientific discovery using a "scientist-in-the-loop" paradigm. It utilizes a coalition of specialized agents that work asynchronously and collaboratively to help domain experts augment their hypothesis generation and experimental planning processes. The specialized agents used by AI co-scientist include:

**Generation Agent:** Generates initial hypotheses and research plans through literature exploration, simulated scientific debates, iterative assumption identification, and research expansion.

**Reflection Agent:** Acts as a scientific peer reviewer, critically assessing hypotheses for correctness, novelty, quality, and safety.

**Ranking Agent:** Employs an Elo-based tournament to rank hypotheses based on pairwise comparisons and scientific debates.

**Proximity Agent:** Calculates hypothesis similarity and builds a proximity graph to aid in de-duplication and idea exploration.

**Evolution Agent:** Refines and improves existing hypotheses by grounding them in literature, combining ideas, simplifying concepts, and out-of-box thinking.

**Meta-review Agent:** Synthesizes insights from all reviews, identifies recurring patterns, and generates meta-reviews to improve the performance of other agents and create research overviews.

Scientists can interact with the AI co-scientist system using natural language to define research goals (including the constraints on the hypothesis and the criteria for evaluating hypothesis quality), provide feedback, and refine hypotheses. The system uses self-play strategies, including a scientific debate and a tournament-based evolution process, to iteratively refine hypotheses and research proposals. It leverages a significant scaling of test-time compute to allow for iterative reasoning, evolution, and improvement of outputs, mimicking the iterative nature of scientific discovery. Additionally, the co-scientist uses various tools, including web search, specialized AI models, domain-specific databases (PubMed) to enhance grounding and the quality of generated hypotheses.

*Supplemental Note 2: Characterization of cell clusters.* In addition to the transcriptomic comparisons with liver cells in the reference databases, differentially expressed genes (DEGs) that were uniquely expressed in each of the cell clusters and their associated biological pathways were identified. For example, DEGs uniquely present in HSC (n=140), MyoF (n=113), Mes^Inh^ (n=50) and Mes^WNT^ (n=246) clusters were identified (**Fig. S4A**). The DEGs in MyoF were most highly correlated with pathways related to growth factor binding, ECM and collagen trimer formation, and the TGFβ response (**Figs. S4B-C, S5**); and microHO MyoF expressed multiple markers that are characteristic of MyoF in human liver (*TIMP1/3, SERPLINE1, LTBP1/2)*. The DEGs in the Mes^Inh^ cells were most highly correlated with negative regulation of peptidase and hydrolase activities, and with ECM degradation. Interestingly, Mes^Inh^ cells express mRNAs encoding proteins that suppressed fibrosis in murine models: *ECM1, PI16 [2], CD47 [3], and CD109 [4]*. Of relevance, ECM1 maintains TGFβ in its inactive form by interacting with αv integrins, which prevents the activation of HSC and liver fibrosis [5]. The DEGs in Mes^WNT^ were most highly correlated with the Wnt/PCP signaling pathway, and were in the Wnt pathway (*LEF1, FOXO3* and *PRICKLE1)*. Although activation of canonical Wnt signaling is required for TGFβ-mediated fibrosis [6], FOXO3 protect against liver fibrosis [7] and PRICKLE1 *(*a WNT/planar cell polarity (PCP) protein) is a negative regulator of the Wnt/beta-catenin pathway [8]. Transcriptomic comparisons with fetal, adult normal and diseased human liver samples identified microHO clusters resembling MyoF and hepatic stellate cells (HSC) in human liver; along with clusters resembling other mesenchymal cell types (Mes1-2) and mesothelial cells (**Fig. S4D**).

There were 234 unique DEGs in Progenitor cells, 269 in EMT, 114 in Cho and 555 in Hep^WNT^ cells (**Fig. S6A**). The Progenitor cell DEGs (*CDK1*, *LEF1, PARP1, RSPO2, TBX20 and DLK1)* were most highly correlated with liver progenitor cells, HDAC binding, Wnt signaling, epithelial proliferation and mitotic pathways (**Fig. S6B-C**). DEGs (*RSPO3, FZD5 and LIFR)* in Hep^WNT^ were most highly correlated with WNT pathway regulation: WNT2 controls cholesterol uptake and bile acid conjugation in hepatocytes through FZD5 [9]; and LIFR promotes liver regeneration [10]. The Cho cell DEGs were most highly correlated with epithelial development and actin binding: PTN regulates the ductal reaction to liver injury [11]; and THBS1 modulates hepatic steatosis in mice and hepatic fat content in humans [12]. EMT cluster DEGs (*NTN4*, *EGFR*, *LAMs* and *CEACAM)* were most highly correlated with ECM organization, MET, ERBB signaling and receptor tyrosine kinase activity. Interestingly, *CEACAM6* is upregulated in response to injury or inflammation and can influence the regeneration of damaged tissues [13], and *EGFR* is required for efficient liver regeneration [14]. When the cluster gene signatures were compared with their *in vivo* counterparts, the MKi67^+^ preCho cells expressed genes with unique functional annotations that resembled bi-potent progenitor cells; Hep1 cells expressed lipid metabolism genes; Progenitor cells resembled fetal liver cells; and EMT cells were enriched with genes characteristic of the epithelial to mesenchyme transition process, and they resembled EPCAM^+^ fetal and adult liver cells. Cho cells had similarities with cholangiocytes; Hep2 had similarities with hepatocyte precursors; and Hep^WNT^ was a differentiated hepatocyte (**Figs. S6D, S7).**

**Methods**

The chemicals, drugs and antibodies used in this study are shown in Tables S1-S4.

*Generation of a collagen reporter iPSC line and microHO generation.* The human iPSC line (C3 COL1A1-P2A-Clover) used in this study was prepared as previously described [15]. The biopsy sample used to generate it was obtained according to a protocol (number 10368) approved by the Institutional Review Board at Stanford. In brief, a clover expression cassette (**P2A-Clover**) was inserted at the COOH terminus of the endogenous *COL1A1* gene of the C3 iPSC line without a STOP codon using a sgRNA (TTGGGATGGAGGGAGTTTAC). To produce microHOs, 50~60% confluent iPSC cultures were cultured in a series of differentiation media as previously described [15]. First, a definitive endoderm differentiation media that consisted of Advanced DMEM/F-12 (Gibco, 12634010) + ITS (Gibco, 41400045) supplemented with 0.1 mM nonessential amino acids, 1 mM pyruvate and 2 mM L-alanyl-L-glutamine dipeptide (GlutaMAX). On days 1 to 2, 100 ng/ml Activin-A (Peprotech, Rocky Hill, NJ), 10 ng/ml BMP4 (Peprotech), 100 ng/ml bFGF (Peprotech), 3 mM CHIR99021 (Sellechchem, Houston, TX) and 10 uM LY294002 (Sellechchem) were added the culture. On day 3, 100 ng/ml Activin-A and 100 ng/ml bFGF were added the definitive endoderm differentiation medium. From days 4 to 9, 20 ng/ml FGF10 (Peprotech) and 20 ng/ml BMP4 were added to the hepatoblast medium, which consisted of which consisted of Advanced RPMI 1640 Medium (Gibco) that was supplemented with GlutaMAX and ITS. After day 9, the hepatoblasts were dissociated to single cells in Accutase (Invitrogen) medium with 10 uM Y-27632 (Santa Cruz Biotechnology, Dallas, Texas). Then, 10,000 cells/well were re-aggregated in low-cell-adhesion Nunclon™ Sphera™ 96 well Microplates (ThermoFisher) that contained serum-free HO growth and differentiation medium, which consisted of William's E medium supplemented with 0.1% Polyvinyl alcohol (Sigma-Aldrich), 0.1 mM nonessential amino acids, 1 mM pyruvate, 2 mM L-alanyl-L-glutamine dipeptide (GlutaMAX), 10 mM Y-27632, 100 ng/ml EGF, 10 ng/ml HGF, 10 mM Dexamethasone and 10 mM Hydrocortisone (Sigma, St. Louis MO). On day 13, 50 ng/ml of TGFβ1 (Peprotech, 100-21; or Sinobiological, 10804-HNAC) and drugs at the indicated concentration were added to each microwell. The microHO cultures were allowed to differentiate over 21 to 31 days.

*Supervised image analysis*. High content screening data was collected from microHOs as previously described [15]. In brief, for the COL1A1:Clover and Hoechst 33342 (Invitrogen, H3570) channels, z-stacked images were captured from day 14 to day 31 cultures with a Molecular Devices ImageXpress Micro Confocal system. For each 10X confocal z-stack field, maximum intensity projections (MIPs) were generated from all acquired z-planes (20 to 40 per microHO). The MIPs from each channel from the same microHO were used for region of interest (ROI) segmentation. Then, 18 features from two channels were extracted using customized FIJI macro code [16] to build the ‘Feature X organoid’ matrix with the features shown in Figure S2 [17]. This matrix is used to compute *Pearson's* correlation coefficients that were used to generate the principal component analyses (PCA) and heatmaps shown. An unpaired Student’s t-test was used to test whether the measurements were significantly different between each comparison group. The macro script used for this analysis is available upon request.

*Flow cytometry.* Single cell suspensions were prepared from microHOs by protease digestion as described [18]. Flow cytometry was performed using at a BD Accuri C6 flow cytometer and the conjugated antibodies listed in Table S4. The data were analyzed using FCS Express (denovosoftware.com) software. A density plot of forward scatter height (FSC-H) vs. forward scatter area (FSC-A) was used to exclude doublets.

*Trichrome staining*. At the experimental end point (between days 20 to 30), the NC, TGFβ1 and TGFβ1 drug treated microHOs were harvested, allowed to settle by gravity, and were embedded in low melting point agarose (IBI Scientific, Dubuque, Iowa). The embedded organoids were then processed by sectioning of paraffin-embedded microHOs to produce 10-micron sections. To assess the amount of collagen in the microHOs, trichrome staining of the sections was performed using the MASSON'S 2000 TRICHROME STAIN KIT according to the manufacturer’s instructions. Image segmentation and quantitative measurement of collagen rich areas was performed using our previously described methods [19]. In brief, Fiji (2.1.0) implementation of ImageJ was used to quantify the areas of positive staining. The ‘Trainable Weka Segmentation’ plug-in was used to train the classifiers and calculate the test experimental image. Statistical analysis was performed using a one-way ANOVA to assess the overall differences between group means, and Tukey's post-test was then used for pairwise comparisons to identify groups with significant differences in their means.

*scRNA-sequencing****.*** Control, TGFβ1 and TGFβ1+drug-treated microHOs (n>30 per group) were harvested from multiple independently prepared sets of cultures, and single cell suspensions were prepared by protease digestion as described [18]. Single cell suspensions were visually inspected under a microscope, cells were counted using a Scepter™ 2.0 Handheld Automated Cell Counter (EMD Millipore, Burlington, MA), and then resuspended in PBS with 0.01% BSA. scRNA-seq libraries were prepared by using the spit-pool based scRNA-seq method (Evercode™ WT mini/WT/Mega from Parse biosciences) according to the manufacturer’s instructions. In brief, 1500/4000/10,000 cells from each group were loaded for the first round of sample barcoding respectively according to which format of kit was used, and the final pooled cells were divided into 2/8/18 equal libraries for sequencing. The expression matrix was generated using the ‘Parse Biosciences analysis pipeline.’ A total of 60,701 features that were generated from 613,826 cells, which passed quality control for processing of the scRNA-seq data obtained from 17 experimental batches.

*scRNA-seq data analysis***.** The scRNA-seq data was imported into ‘Seurat’ [20] for the

subsequent analysis steps. (i) Cells with unique gene counts <200 or >7000 or where the percentage of mitochondrial mRNAs was >25% were removed. (ii) The 17 batches of data were integrated by ‘RPCA’ processing methods using the Seurat integration function. (iii) 30 principal components were used to construct the shared nearest neighbor (SNN) graph, and the parameter regulating the resolution of the ‘Find Clusters’ program, was set to 0.5. This resulted in the identification of 17 unique clusters for all 613,826 cells. To identify the cell type of each cluster, differentially expressed genes for the pre-defined cell types were computed using the ‘FindAllMarkers’ function within the Seurat Package with the following parameters: only.pos = F, min.pct = 0.25, logfc.threshold = 0.25. Seurat then identifies the differentially expressed genes using the non-parametric Wilcoxon rank sum test. The top 50 upregulated DEGs were used for the GO biological process analysis, which was performed using the ‘clusterProfiler’ for GO over-representation analysis [21].

*Module score calculation*. Module scores were calculated to assess the relationship between the transcriptomes of microHO clusters and the different types of cells in human liver tissue. scRNA-seq data obtained from normal and cirrhotic human liver tissue (GSE136103) [22] was used to identify the different types of mesenchymal cells in liver (myofibroblast, hepatic stellate cell (HSC), vascular smooth muscle cell (VSMC) and mesothelia (Meso)). The differentially expressed genes for each mesenchymal cell type were used to calculate module score for each of the clusters in the microHOs. A subset of the HSC scRNA-seq data obtained from GSE212837 [23] was used to identify the normal and NASH HSC types. A subset of the mesenchymal cell scRNA-seq data obtained from GSE185477 [24] was used to identify normal HSCs, myofibroblasts (FB) and VSMC. For the epithelial cell clusters, scRNA-seq data obtained from GSE130473 [25] was used to identify the subpopulations of hepatocytes and cholangiocytes in fetal and adult liver. Also, hepatic lineage cells from different developmental stages in the human liver scRNA-seq data obtained from CRA002443 [26] was used to identify cell lineages in fetal liver. A subset of the cholangiocyte scRNA-seq data obtained from GSE185477 [24] was used to identify normal hepatic progenitors and the subpopulations of cholangiocytes.

*Cell type annotation*. R package Azimuth [27] was used to annotate microHO scRNA-seq data to the reference dataset. Azimuth uses a 'reference-based mapping' pipeline that inputs a count matrix; and then performs the normalization, visualization, cell annotation, and assesses differential expression of the data. This human reference consists of 79,492 integrated liver cells from data collected in 5 different studies [22, 28].

*Cell status analysis*. The ‘Cellular potency categories’ and the absolute developmental potential were assessed using ‘CytoTRACE 2’ [29] and the microHO scRNA-seq data. The predicted ‘potency score’ provide a continuous measure of developmental potential; and they range from 0 (differentiated) to 1 (totipotent). The raw count from the Seurat object is used to directly to compute CytoTRACE score. ‘RNA velocity’ analysis infers the cell state by measuring the ratio of un-spliced to spliced mRNA transcripts, which provides information about the transcriptional activity of a gene and its direction. The transcript assignment file (tscp_assignment.csv.gz from the Parse pipeline output directory) contains the splicing information for each transcript identified in a Parse assay. The splicing information is used to generate the splice matrices required to run scVelo [30]. The Anndata file, which is required for scVelo, was generated using the Seurat object that contains the metadata, and splicing matrix. RNA velocity was estimated with the ‘stochastic model’ (using second-order moments).

*Cluster over representation analysis*. The pathways associated with the DEGs in microHO cell clusters was performed using the ‘clusterProfiler’ package [21]. The top 100 DEGs, which were ranked by their ‘avg_log2FC’ using the Seurat ‘FindAllMarkers’ function, are the input gene list for calculating the over-representation analysis (ORA). The databases used in this study are: Gene Ontology (GO, <http://www.geneontology.org>); Kyoto Encyclopedia of Genes and Genomes (KEGG, <https://www.genome.jp/kegg/>); ReactomePA (<http://bioconductor.org/packages/ReactomePA>); and the Molecular Signatures Database (MSigDb, <http://software.broadinstitute.org/gsea/msigdb>).

*Cell type Differential composition analysis*. The one-way ANOVA was used to compare the means for each cell type percentage between the different drug treatment groups. In brief,

Dunnett's test was used as a post-hoc test after a one-way ANOVA to compare the means of multiple treatment to a TGFβ treatment. We also use ‘sccomp’[31] to test differences in cell type proportions and variability. In total we have 17 batch experiments, 16 cell types and 8 treatments were included for differential composition analysis.

*snATAC-seq*. snATAC-seq used the same single cell samples as those prepared for scRNA-seq. For the snATAC-seq analyses, 3 batches of microHO samples (24 samples) were prepared. Single nuclei were isolated from those samples using the nuclei Isolation kit (Nuclei EZ Prep, Sigma Aldrich, NUC101-1KT) according to the manufacturer’s instructions and the samples were pre-indexed using the scATAC Pre-Indexing Kit (ScaleBio, San Diego, CA) according to the manufacturer’s instructions. The pooled single nuclei were loaded onto the 10xChromium Next GEM Single Cell v2 workflow.

*snATAC-seq Data Processing and analysis***.** The raw sequencing data were demultiplexed, and FASTQ files were generated using the the ScaleTagToolkit from the Scale Bio seq Suite Suite (<https://github.com/ScaleBio/ScaleTagToolkit>). This suite processes the data from demultiplex samples, and the 150 bp paired-end (PE) snATAC-seq reads were aligned to the GRCh38 (hg38) reference genome. The mapped Tn5 insertion sites (fragments.tsv files) from scaletagtoolkit were read into the ArchR (v1.0.3) R package [32]. To ensure high-quality sequencing and signal-to-background ratio, cells with fewer than 1,000 unique fragments or TSS enrichment below 4 were filtered out. After filtering, there were 34,698 nuclei with an average of 7000 reads per nuclei. To calculate TSS enrichment, the genome-wide Tn5-corrected insertions were aggregated ±2,000 bp relative to each TSS (TSS-strand-corrected). This profile was normalized to the mean accessibility ±1,900–2,000 bp from the TSS, smoothed every 51 bp, and the maximum smoothed value was reported as TSS enrichment in R.

The Latent Semantic Indexing (LSI) dimensionality reduction was performed by appending fragment files from all 24 samples of microHOs analyzed. Chromatin-derived gene accessibility scores were computed by aggregating the snATAC-seq reads for each cell, which were weighted by their distance from each gene within its cis-regulatory domain. The Leiden clustering algorithm [33] was used to identify cell clusters in the LSI subspace. Canonical correlation analysis (CCA), implemented in Seurat [20], was used to align and match cells from the scRNA-seq and snATAC-seq experiments. Log2-transformed gene accessibility scores served as proxies for gene expression in snATAC-seq cells. For integration, we selected the 2,000 most variable genes from each modality and used Seurat’s FindTransferAnchors() function with CCA as the reduction method and k.anchor = 10. Each scRNA-seq cell was mapped to its nearest neighbor in snATAC-seq using a nearest-neighbor search in the joint CCA L2 space. Nearest neighbors were identified with the FNN R package ([FNN](https://rdrr.io/cran/FNN/)), using the ‘kd_tree’ algorithm and Euclidean distance. The labels from the scRNA-seq data were transferred to snATAC-seq data for chromatin accessibility analysis. Differential gene analysis was performed using the getMarkerFeatures function in ArchR, based on the gene scores computed from chromatin accessibility near gene coordinates. Differential genes between different treatment conditions and normal control cells were identified using FDR ≤ 0.05 and Log2FC ≥ 0.25 thresholds. To calculate the pseudotime and to construct the cellular differentiation trajectory for mesenchymal, epithelial clusters, the HSC and Progenitor clusters were set as starting point for each group, respectively.

*EpiTrace analysis***.** A count matrix was used where; the rows are genomic loci, and the columns are cells; and elements are ATAC-seq read numbers and a data frame or GRanges object corresponds to the genomic loci were extracted from ArchR object, the ‘EpiTraceAge_Convergence’ function was used to infer age from single cells [34].

*Statistical analysis*.

For high content imaging and Trichrome stained images analyses, each time point used > 3 batches, and each batch had > 8 microHOs. An unpaired Student’s t-test was used to test whether the measurements were significantly different between a treatment group and the TGFβ group. The data shown in **Fig. 1H, 1J, 2B, 2D, 2F** and **S2D-J** were analyzed using an unpaired, two-tailed Student's t-test. For analysis of the scRNA-seq data, the results of 17 experimental batches, 16 identified cell types and 8 different treatments were included for the differential cell composition analyses. The number of cells and experimental batches analyzed to generate the scRNA-Seq data, and the ANOVA and Dunnet statistics for the clusters are described in supplemental data files **S2A-E**; **S2F** shows the number of single nuclei and the median number of fragments for each of the 24 samples used for snATAC-seq; **S2G** shows the list of genes from ‘differential gene score’ calculated from the snATAC-seq data organized by cell cluster. In **Figs. 3I** and **5A**, a one-way ANOVA was used to compare the means obtained for each cell type percentage between the different drug treatment groups. In brief, Dunnett's test was used as a post-hoc test after a one-way ANOVA to compare the effects of multiple treatments on the mean value after TGFβ treatment, and that in **Fig. 6G-H** were analyzed using the Wilcoxon test. ‘sccomp’ [31] was used to test differences in cell type proportions and variability. The statistical significance for all tests were indicated as follows: ns = not significant; * = p-value<0.05; ** = p-value<0.001; *** = p-value<0.001; and **** = p-value<0.0001. All data are presented as mean + SEM. All statistical analyses were plotted and performed using the R package programs: rstatix, ggpubr and ggplot2.

**Table S1.** Information about the drugs examined in this study.

**Table S2.** Chemicals and reagents used in this study.

**Table S3**. The list of primary antibodies used in this study.

**Table S4**. The conjugated antibodies used in this study were all obtained from Biolegend.

**Supplemental References**

[1] J. Gottwies, W.-H. Weing, A. Daryin, T. Tu, A. Palepu, P. Sirkovic, A. Myaskovsky, F. Weissenberger, K. Rong, R. Tanno, K. Saab, D. Popovici, J. Blum, F. Zhang, K. Chou, A. Hassidim, B. Gokturk, A. Vahdat, P. Kohli, Y. Matias, A. Carroll, K. Kulkarni, N. Tomasev, V. Dhillon, D. Vaishnav, B. Lee, T. R. D. Costa, J. R. Penades, G. Peltz, Y. Xu, A. Pawlosky, A. Karthikesalingam, V. Natarajan, *arXiv:2502.18864v1* **2025**, <https://doi.org/https://arxiv.org/pdf/2502.18864>.

[2] M. Deng, S. Yang, Y. Ji, Y. Lu, M. Qiu, Y. Sheng, W. Sun, X. Kong, *J Cell Mol Med* **2020**, *24* (9), 5249, <https://doi.org/10.1111/jcmm.15178>.

[3] H. C. Tao, K. X. Chen, X. Wang, B. Chen, W. O. Zhao, Y. Zheng, Y. G. Yang, *Front Immunol* **2020**, *11*, 148, <https://doi.org/10.3389/fimmu.2020.00148>.

[4] H. Naoi, Y. Suzuki, A. Miyagi, R. Horiguchi, Y. Aono, Y. Inoue, H. Yasui, H. Hozumi, M. Karayama, K. Furuhashi, N. Enomoto, T. Fujisawa, N. Inui, S. Mii, M. Ichihara, M. Takahashi, T. Suda, *J Immunol* **2024**, *212* (7), 1221, <https://doi.org/10.4049/jimmunol.2300285>.

[5] W. Fan, T. Liu, W. Chen, S. Hammad, T. Longerich, I. Hausser, Y. Fu, N. Li, Y. He, C. Liu, Y. Zhang, Q. Lian, X. Zhao, C. Yan, L. Li, C. Yi, Z. Ling, L. Ma, X. Zhao, H. Xu, P. Wang, M. Cong, H. You, Z. Liu, Y. Wang, J. Chen, D. Li, L. Hui, S. Dooley, J. Hou, J. Jia, B. Sun, *Gastroenterology* **2019**, *157* (5), 1352, <https://doi.org/10.1053/j.gastro.2019.07.036>.

[6] A. Akhmetshina, K. Palumbo, C. Dees, C. Bergmann, P. Venalis, P. Zerr, A. Horn, T. Kireva, C. Beyer, J. Zwerina, H. Schneider, A. Sadowski, M. O. Riener, O. A. MacDougald, O. Distler, G. Schett, J. H. Distler, *Nature communications* **2012**, *3*, 735, <https://doi.org/10.1038/ncomms1734>.

[7] P. Piccolo, R. Ferriero, A. Barbato, S. Attanasio, M. Monti, C. Perna, F. Borel, P. Annunziata, A. Carissimo, R. De Cegli, L. Quagliata, L. M. Terracciano, C. Housset, J. H. Teckman, C. Mueller, N. Brunetti-Pierri, *Proc Natl Acad Sci U S A* **2021**, *118* (10), <https://doi.org/10.1073/pnas.2025242118>.

[8] D. W. Chan, C. Y. Chan, J. W. Yam, Y. P. Ching, I. O. Ng, *Gastroenterology* **2006**, *131* (4), 1218, <https://doi.org/10.1053/j.gastro.2006.07.020>.

[9] E. Kaffe, M. Roulis, J. Zhao, R. Qu, E. Sefik, H. Mirza, J. Zhou, Y. Zheng, G. Charkoftaki, V. Vasiliou, D. F. Vatner, W. Z. Mehal, AlcHepNet, K. Yuval, R. A. Flavell, *Cell* **2023**, *186* (18), 3793, <https://doi.org/10.1016/j.cell.2023.07.017>.

[10] Y. Deng, Z. Zhao, M. Sheldon, Y. Zhao, H. Teng, C. Martinez, J. Zhang, C. Lin, Y. Sun, F. Yao, M. A. Curran, H. Zhu, L. Ma, *Nat Metab* **2024**, *6* (9), 1756, <https://doi.org/10.1038/s42255-024-01110-y>.

[11] G. A. Michelotti, A. Tucker, M. Swiderska-Syn, M. V. Machado, S. S. Choi, L. Kruger, E. Soderblom, J. W. Thompson, M. Mayer-Salman, H. A. Himburg, C. A. Moylan, C. D. Guy, K. S. Garman, R. T. Premont, J. P. Chute, A. M. Diehl, *Gut* **2016**, *65* (4), 683, <https://doi.org/10.1136/gutjnl-2014-308176>.

[12] J. Bai, M. Xia, Y. Xue, F. Ma, A. Cui, Y. Sun, Y. Han, X. Xu, F. Zhang, Z. Hu, Z. Liu, Y. Liu, G. Cai, W. Su, X. Sun, H. Wu, H. Yan, X. Chang, X. Hu, H. Bian, P. Xia, J. Gao, Y. Li, X. Gao, *EBioMedicine* **2020**, *57*, 102849, <https://doi.org/10.1016/j.ebiom.2020.102849>.

[13] S. E. Lin, A. M. Barrette, C. Chapin, L. W. Gonzales, R. F. Gonzalez, L. G. Dobbs, P. L. Ballard, *Physiol Rep* **2015**, *3* (12), <https://doi.org/10.14814/phy2.12657>.

[14] A. Natarajan, B. Wagner, M. Sibilia, *Proc Natl Acad Sci U S A* **2007**, *104* (43), 17081, <https://doi.org/10.1073/pnas.0704126104>.

[15] Y. Guan, Z. Fang, A. Hu, W. Ren, P. K. Johansson, S. C. Heilshorn, A. Enejder, G. Peltz, *JCI Insight* **2025**, *10* (2), e187099, <https://doi.org/https://doi.org/10.1172/jci.insight.187099>.

[16] J. Schindelin, I. Arganda-Carreras, E. Frise, V. Kaynig, M. Longair, T. Pietzsch, S. Preibisch, C. Rueden, S. Saalfeld, B. Schmid, J. Y. Tinevez, D. J. White, V. Hartenstein, K. Eliceiri, P. Tomancak, A. Cardona, *Nat Methods* **2012**, *9* (7), 676, <https://doi.org/10.1038/nmeth.2019>.

[17] I. Lukonin, D. Serra, L. Challet Meylan, K. Volkmann, J. Baaten, R. Zhao, S. Meeusen, K. Colman, F. Maurer, M. B. Stadler, J. Jenkins, P. Liberali, *Nature* **2020**, *586* (7828), 275, <https://doi.org/10.1038/s41586-020-2776-9>.

[18] Y. Guan, D. Xu, P. M. Garfin, U. Ehmer, M. Hurwitz, G. Enns, S. Michie, M. Wu, M. Zheng, T. Nishimura, J. Sage, G. Peltz, *JCI Insight* **2017**, *2* (17), pii: 94954, <https://doi.org/10.1172/jci.insight.94954>.

[19] Y. Guan, A. Enejder, M. Wang, Z. Fang, L. Cui, S. Y. Chen, J. Wang, Y. Tan, M. Wu, X. Chen, P. K. Johansson, I. Osman, K. Kunimoto, P. Russo, S. C. Heilshorn, G. Peltz, *Nature communications* **2021**, *12* (1), 6138, <https://doi.org/10.1038/s41467-021-26410-9>.

[20] A. Butler, P. Hoffman, P. Smibert, E. Papalexi, R. Satija, *Nat Biotechnol* **2018**, *36* (5), 411, <https://doi.org/10.1038/nbt.4096>.

[21] T. Wu, E. Hu, S. Xu, M. Chen, P. Guo, Z. Dai, T. Feng, L. Zhou, W. Tang, L. Zhan, X. Fu, S. Liu, X. Bo, G. Yu, *Innovation (Camb)* **2021**, *2* (3), 100141, <https://doi.org/10.1016/j.xinn.2021.100141>.

[22] P. Ramachandran, R. Dobie, J. R. Wilson-Kanamori, E. F. Dora, B. E. P. Henderson, N. T. Luu, J. R. Portman, K. P. Matchett, M. Brice, J. A. Marwick, R. S. Taylor, M. Efremova, R. Vento-Tormo, N. O. Carragher, T. J. Kendall, J. A. Fallowfield, E. M. Harrison, D. J. Mole, S. J. Wigmore, P. N. Newsome, C. J. Weston, J. P. Iredale, F. Tacke, J. W. Pollard, C. P. Ponting, J. C. Marioni, S. A. Teichmann, N. C. Henderson, *Nature* **2019**, *575* (7783), 512, <https://doi.org/10.1038/s41586-019-1631-3>.

[23] S. Wang, K. Li, E. Pickholz, R. Dobie, K. P. Matchett, N. C. Henderson, C. Carrico, I. Driver, M. Borch Jensen, L. Chen, M. Petitjean, D. Bhattacharya, M. I. Fiel, X. Liu, T. Kisseleva, U. Alon, M. Adler, R. Medzhitov, S. L. Friedman, *Sci Transl Med* **2023**, *15* (677), eadd3949, <https://doi.org/10.1126/scitranslmed.add3949>.

[24] T. S. Andrews, J. Atif, J. C. Liu, C. T. Perciani, X. Z. Ma, C. Thoeni, M. Slyper, G. Eraslan, A. Segerstolpe, J. Manuel, S. Chung, E. Winter, I. Cirlan, N. Khuu, S. Fischer, O. Rozenblatt-Rosen, A. Regev, I. D. McGilvray, G. D. Bader, S. A. MacParland, *Hepatol Commun* **2022**, *6* (4), 821, <https://doi.org/10.1002/hep4.1854>.

[25] J. M. Segal, D. Kent, D. J. Wesche, S. S. Ng, M. Serra, B. Oules, G. Kar, G. Emerton, S. J. I. Blackford, S. Darmanis, R. Miquel, T. V. Luong, R. Yamamoto, A. Bonham, W. Jassem, N. Heaton, A. Vigilante, A. King, R. Sancho, S. Teichmann, S. R. Quake, H. Nakauchi, S. T. Rashid, *Nature communications* **2019**, *10* (1), 3350, <https://doi.org/10.1038/s41467-019-11266-x>.

[26] X. Wang, L. Yang, Y. C. Wang, Z. R. Xu, Y. Feng, J. Zhang, Y. Wang, C. R. Xu, *Cell Res* **2020**, *30* (12), 1109, <https://doi.org/10.1038/s41422-020-0378-6>.

[27] Y. Hao, S. Hao, E. Andersen-Nissen, W. M. Mauck, 3rd, S. Zheng, A. Butler, M. J. Lee, A. J. Wilk, C. Darby, M. Zager, P. Hoffman, M. Stoeckius, E. Papalexi, E. P. Mimitou, J. Jain, A. Srivastava, T. Stuart, L. M. Fleming, B. Yeung, A. J. Rogers, J. M. McElrath, C. A. Blish, R. Gottardo, P. Smibert, R. Satija, *Cell* **2021**, *184* (13), 3573, <https://doi.org/10.1016/j.cell.2021.04.048>.

[28] a) N. Aizarani, A. Saviano, Sagar, L. Mailly, S. Durand, J. S. Herman, P. Pessaux, T. F. Baumert, D. Grun, *Nature* **2019**, *572*, 199, <https://doi.org/10.1038/s41586-019-1373-2>; b) S. A. MacParland, J. C. Liu, X. Z. Ma, B. T. Innes, A. M. Bartczak, B. K. Gage, J. Manuel, N. Khuu, J. Echeverri, I. Linares, R. Gupta, M. L. Cheng, L. Y. Liu, D. Camat, S. W. Chung, R. K. Seliga, Z. Shao, E. Lee, S. Ogawa, M. Ogawa, M. D. Wilson, J. E. Fish, M. Selzner, A. Ghanekar, D. Grant, P. Greig, G. Sapisochin, N. Selzner, N. Winegarden, O. Adeyi, G. Keller, G. D. Bader, I. D. McGilvray, *Nature communications* **2018**, *9* (1), 4383, <https://doi.org/10.1038/s41467-018-06318-7>; c) V. L. Payen, A. Lavergne, N. Alevra Sarika, M. Colonval, L. Karim, M. Deckers, M. Najimi, W. Coppieters, B. Charloteaux, E. M. Sokal, A. El Taghdouini, *JHEP Rep* **2021**, *3* (3), 100278, <https://doi.org/10.1016/j.jhepr.2021.100278>; d) M. Zhang, H. Yang, L. Wan, Z. Wang, H. Wang, C. Ge, Y. Liu, Y. Hao, D. Zhang, G. Shi, Y. Gong, Y. Ni, C. Wang, Y. Zhang, J. Xi, S. Wang, L. Shi, L. Zhang, W. Yue, X. Pei, B. Liu, X. Yan, *J Hepatol* **2020**, *73* (5), 1118, <https://doi.org/10.1016/j.jhep.2020.05.039>.

[29] M. Kang, J. J. A. Armenteros, G. S. Gulati, R. Gleyzer, S. Avagyan, E. L. Brown, W. Zhang, A. Usmani, N. Earland, Z. Wu, J. Zou, R. C. Fields, D. Y. Chen, A. A. Chaudhuri, A. M. Newman, *bioRxiv* **2024**, <https://doi.org/10.1101/2024.03.19.585637>.

[30] V. Bergen, M. Lange, S. Peidli, F. A. Wolf, F. J. Theis, *Nat Biotechnol* **2020**, *38* (12), 1408, <https://doi.org/10.1038/s41587-020-0591-3>.

[31] S. Mangiola, A. J. Roth-Schulze, M. Trussart, E. Zozaya-Valdes, M. Ma, Z. Gao, A. F. Rubin, T. P. Speed, H. Shim, A. T. Papenfuss, *Proc Natl Acad Sci U S A* **2023**, *120* (33), e2203828120, <https://doi.org/10.1073/pnas.2203828120>.

[32] J. M. Granja, M. R. Corces, S. E. Pierce, S. T. Bagdatli, H. Choudhry, H. Y. Chang, W. J. Greenleaf, *Nat Genet* **2021**, *53* (3), 403, <https://doi.org/10.1038/s41588-021-00790-6>.

[33] V. A. Traag, L. Waltman, N. J. van Eck, *Sci Rep* **2019**, *9* (1), 5233, <https://doi.org/10.1038/s41598-019-41695-z>.

[34] Y. Xiao, W. Jin, L. Ju, J. Fu, G. Wang, M. Yu, F. Chen, K. Qian, X. Wang, Y. Zhang, *Nat Biotechnol* **2025**, *43* (5), 784, <https://doi.org/10.1038/s41587-024-02241-z>.

**Supplemental Figures**

**
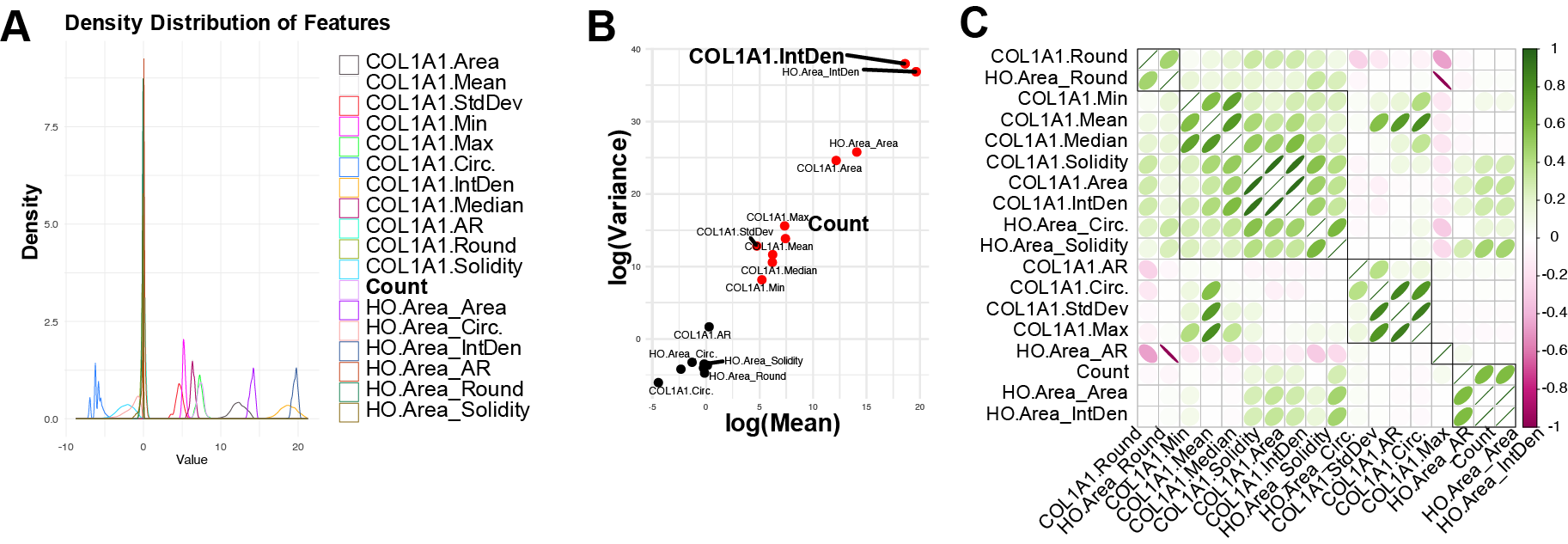
**

**Figure S1**. High content screening (HCS) for pro-fibrotic agents using microHOs.

(**A**) This density plot shows the data distribution for the 18 features extracted from the HCS data. (**B**) This scatter plot shows the mean versus the variance for the 18 features extracted from HCS data. A red color indicates that the log(variance) is >5-fold above the threshold.

(**C**) A heatmap formed using the Pearson's correlation coefficients calculated from the 18 features (shown on each axis) generated from the COL1A1:Clover and nuclear (Hoechst 33342) staining channels, which were obtained from all microHOs and conditions that were examined in this paper. The bar on the right shows the extent of the correlation (by color) shown in each square of the diagram (green is positive, magenta is negative). As can be seen, the features obtained from same channel cluster together. However, features measured using different channels will occasionally cluster together, which indicates that those features were similarly affected by the drugs.

**
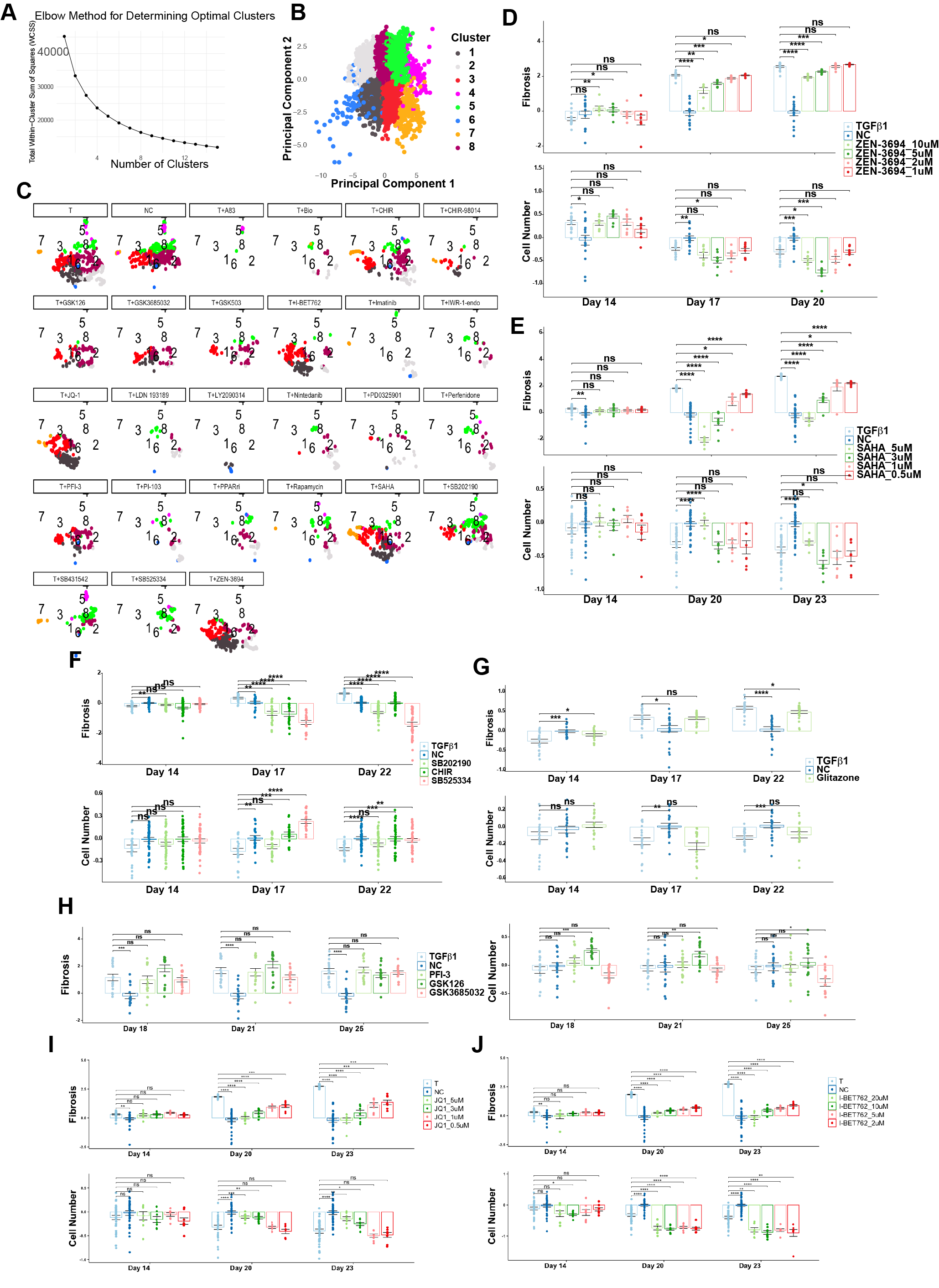
**

**Figure S2**. High content screening for anti-fibrotic agents using microHOs. **A**) This plot shows the elbow method for determining optimal k-mean clustering number for the anti-fibrotic drug datasets. (**B**) This PCA plot shows the clusters generated from the anti-fibrotic datasets. (**C**) This plot shows the clusters projected on a UMAP embedding of anti-fibrotic datasets separated based upon the indicated drug treatments. (**D**, **E**) microHOs were treated with either normal media (NC), 50 ng/mL TGFβ, or TGFβ plus the indicated concentration of ZEN-3694 (**D**) or SAHA (**E**) on day 13. The extent of fibrosis (COL1A1 fluorescence) and cell numbers were serially measured on days 14 through 23. Sixteen microHOs per treatment were assessed per condition. The fibrosis and cell number measurements were normalized relative to NC microHOs. (**F**) microHOs were treated with either normal media (NC); 50 ng/mL TGFβ; or TGFβ with 10 μM of either p38 (SB202190) 3 μM GSK3β (CHIR-99021) or TGFBR1 (SB525334) inhibitor on day 13. The fluorescent signal from COL1A1^+^ cells and the total number of cell nuclei within a microHO were serially measured on days 14 through 22. (**G**) TGFβ-induced fibrosis in microHOs is not blocked by PPARγ inhibitors. microHOs were treated with either normal media (NC), 50 ng/mL TGFβ, or TGFβ and Glitazone (10 μM) on day 13. The fluorescent signal from COL1A1^+^ cells and the total number of cell nuclei within a microHO were serially measured on days 14 through 22. (**H**) TGFβ-induced fibrosis in microHOs is not blocked by SMARCA2/4, EZH2 or DNMT1 inhibitors. microHOs were treated with either normal media (NC), 50 ng/mL TGFβ, or TGFβ and either 10 μM SMARCA2/4 (PFI-3), 10 μM EZH2 (GSK126) or 10 μM DNMT1 (GSK3685032) inhibitor on day 13. The fluorescent signal from COL1A1^+^ cells and the total number of cell nuclei within a microHO were serially measured on days 18 through 25, and 16 microHOs were assessed per condition analyzed. The DNMT1 inhibitor caused significant toxicity. ns, not significant; *, p-value<0.05; **, p-value<0.001; ***, p-value<0.001; and ****p-value<0.0001. (**I-J**) TGFβ-induced fibrosis in microHOs is blocked by BRD4 inhibitors. microHOs were treated with either normal media (NC), 50 ng/mL TGFβ, or TGFβ and the indicated concentration of JQ-1 (**I**) or I-BET762 (**J**). The fluorescent signal from COL1A1^+^ cells and the total number of cell nuclei within a microHO were serially measured on days 14 through 23, and 32 microHOs were assessed per condition analyzed. ns, not significant; *, p-value<0.05; **, p-value<0.001; ***, p-value<0.001; and ****p-value<0.0001.


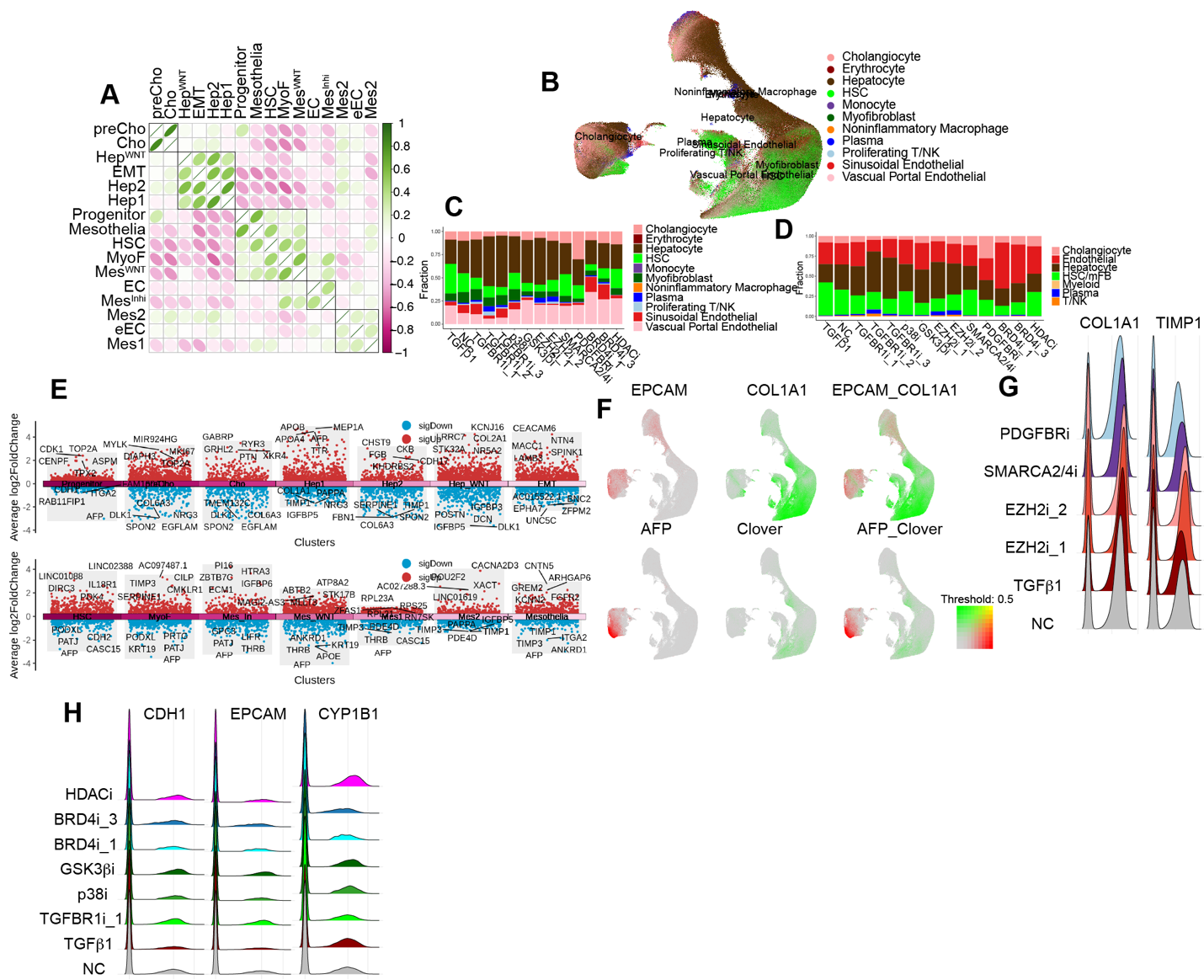


**Figure S3**. (**A**) A heatmap showing the Pearson correlation coefficients calculated for comparing the average transcriptome of each of the 16 cell clusters using the scRNA-seq data described in Figure 2A. The bar on the right shows the extent of the correlation (by color) that is shown in each square of the diagram (green is positive, magenta is negative). Note that the mesenchymal cells cluster together and are separated from the epithelial cell types, and vice versa. (**B-D**) Cell type annotation of the clusters in the scRNA-seq data was determined using the Azimuth liver reference. These the liver cell annotations (L2) were projected onto the UMAP of the microHOs (B). The percentage of each predicted cell type (L1 and L2), which represents the average obtained from analyses performed on ten batches of microHOs, were used to generate the scRNA-seq data for each indicated condition (C-D). (**E**) A volcano plot showing the top 5 differentially expressed genes among the 16 cell clusters. As examples: the hepatocyte cluster (Hep1) expresses *AFP, TTR, CDH1(E-Cadherin)* and *APOB* mRNAs; the endothelial cell cluster (eEC and EC) express *PECAM1* mRNA; and the myofibroblast cluster (MyoF) expresses *TIMP3, COL1A1* and *Clover* mRNAs. (**F**) A blended feature plot showing the epithelial and mesenchymal cells in microHOs, which are separated based upon *EPCAM* vs *COL1A1* or *AFP* vs *Clover* mRNA expression. (**G**) Ridge plots shows MyoF population markers (*COL1A1^+^, TIMP1^+^*) are not affected by EZH2i, SMARCA2/4i and PDGFRBi treatment in TGFβ microHOs.

(**H**) Ridge plots shows epithelial population marker (*CDH1, EPCAM and CYP1B1)* expression is restored by when the indicated drugs are co-administered with TGFβ.


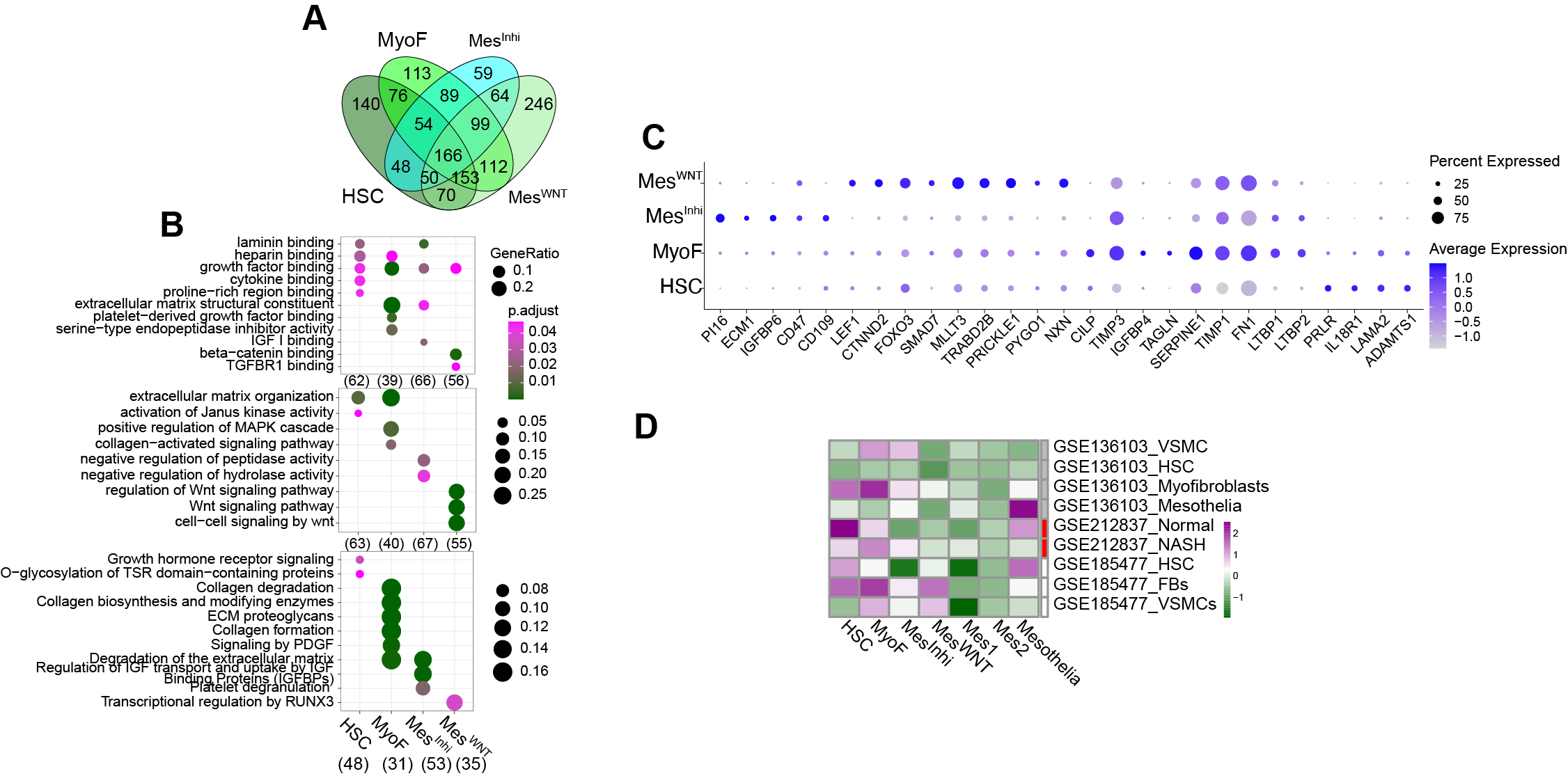


**Figure S4.** Analysis of mesenchymal clusters. (**A**) This Venn Diagram shows number of differentially expressed or overlapping genes in the hepatic stellate cell (HSC), myofibroblast (MyoF), Mes^Inhi^ and Mes^WNT^ clusters. (**B**) Over Representation Analysis (ORA) for 4 mesenchymal clusters present in microHOs. The unique DEGs for each cell type were annotated using the GO-MF, GO-BF and Reactome databases. (**C**) Dot plots showing the average level of expression and the percentage of cells expressing the indicated DEGs for each mesenchymal clusters. (**D**) The relationship between the transcriptomes of the seven mesenchymal clusters (HSC, MyoF, Mes^Inhi^, Mes^WNT^, Mes1-2 and Mesothelia) in microHOs and human liver disease cell types found in 3 reference datasets was assessed by calculating a module score. The datasets contain HSC in normal and cirrhotic human livers, vascular smooth muscle cells (VSMC), MyoF, and mesothelial cells in GSE136103; mesenchymal cells in normal liver and NASH liver (GSE212837); and HSC, VSMC and MyoF (FB) in GSE185477.

**
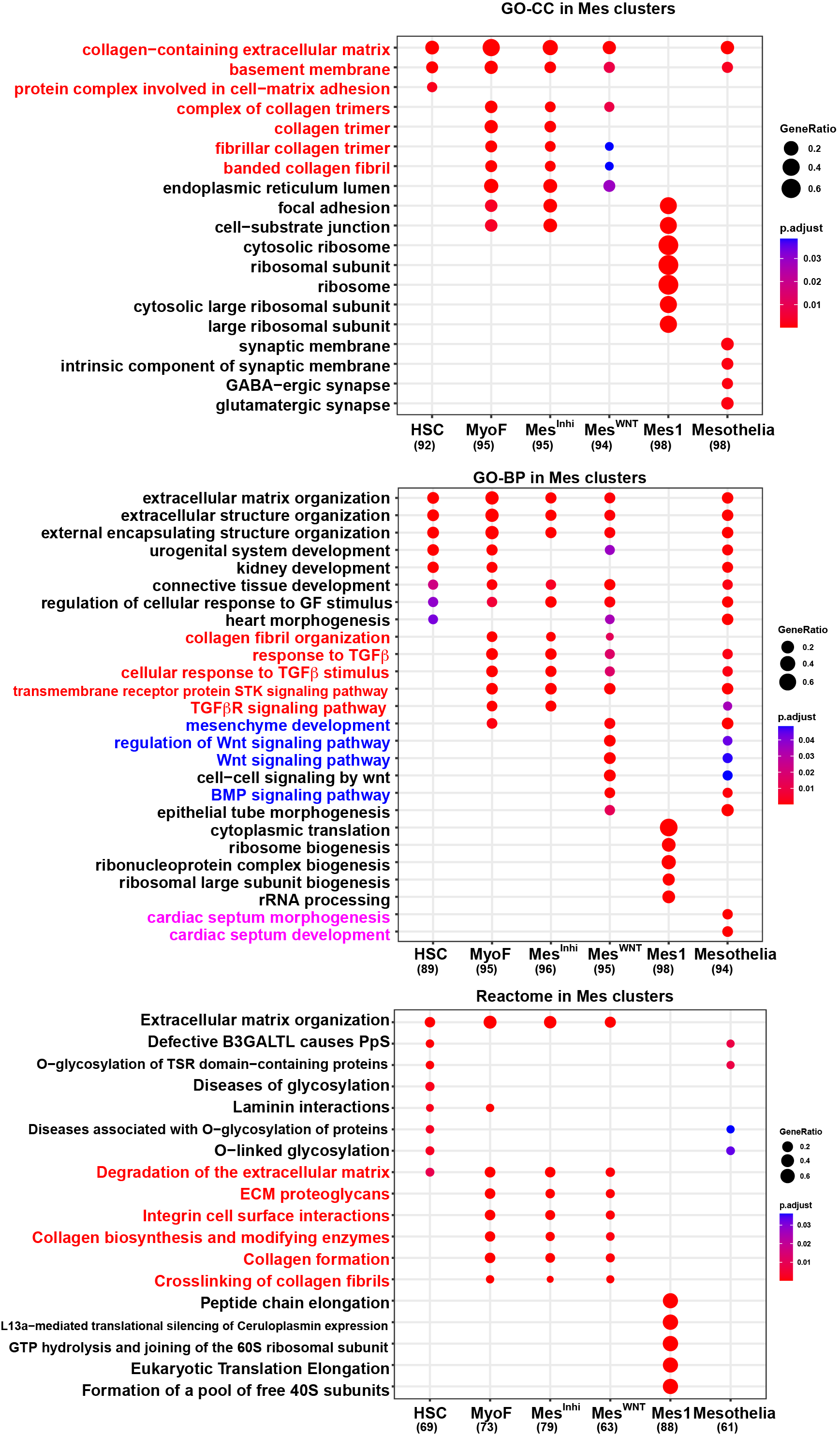
**

**Figure S5.** Pathway enrichment analysis for six mesenchymal clusters present in microHOs. The pathways associated with the top 100 DEGs for each cell type were annotated using the GO-CC, GO-BF and Reactome databases. The insets on the right show the gene ratio, which represents the proportion of the genes in the input gene list that are associated with a particular term; and the color-coded adjusted p-value for the statistical significance of the enrichment for a particular term in each cluster of genes.


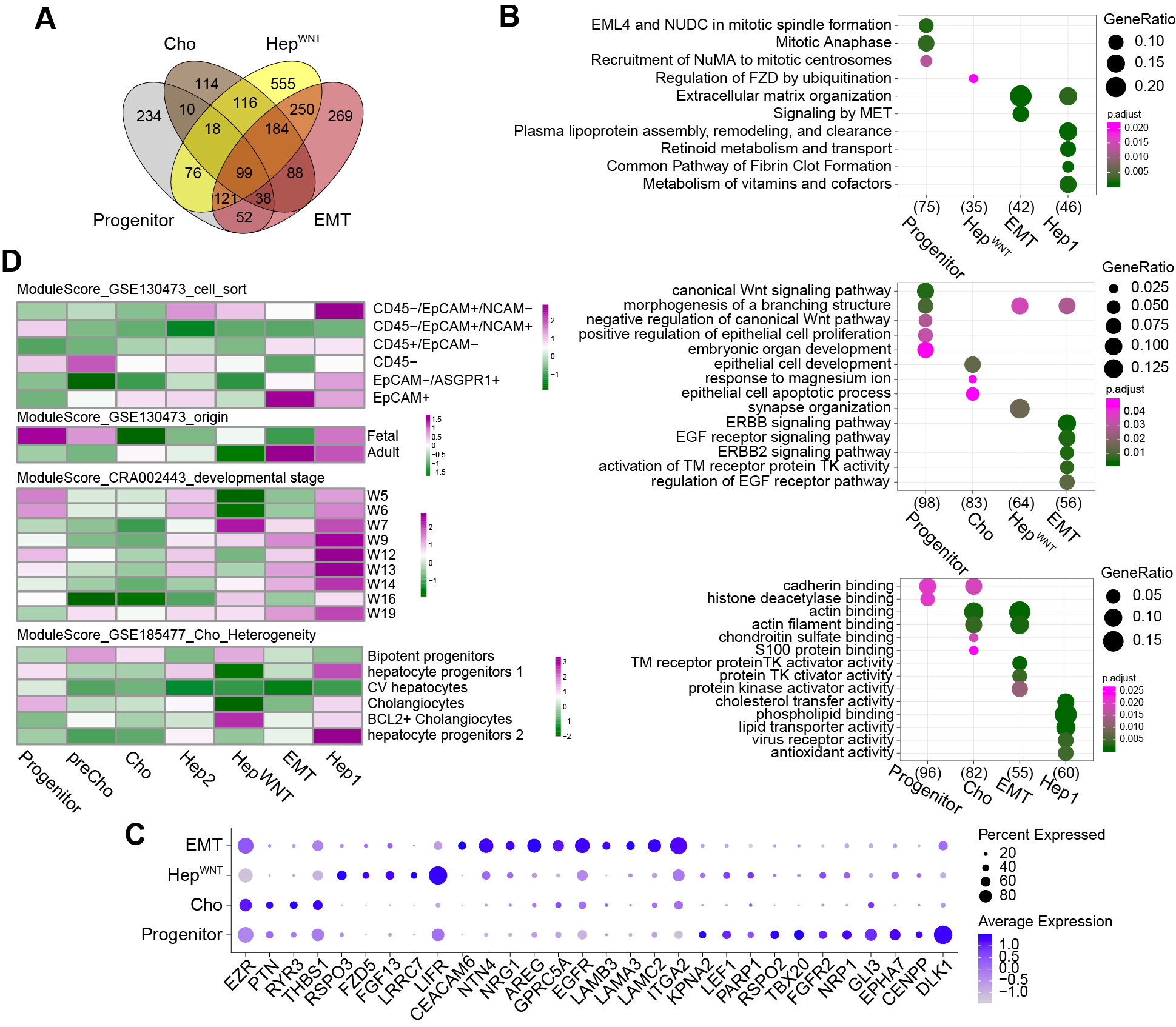


**Figure S6.** Analysis of epithelial clusters. (**A**) This Venn Diagram shows number of differentially expressed or overlapping genes in the hepatic progenitor, EMT, Cho and Hep^WNT^ clusters. (**B**) ORA for 4 epithelial clusters present in microHOs. The unique DEGs for each cell type were annotated using the GO-MF, GO-BF and Reactome databases. (**C**) Dot plots showing the average level of expression and the percentage of cells expressing the indicated DEGs for epithelial clusters. (**D**) Module scores assess the relationship between the transcriptomes of the seven epithelial clusters (Progenitor, preCho, Cho, EMT, Hep^WNT^, Hep1-2) in microHOs with human fetal and adult liver cell types found in 3 reference datasets: hepatobiliary hybrid progenitors in fetal liver (GSE130473); hepatogenesis in different developmental stages of fetal liver (CRA002443); or various types of cholangiocytes in human liver (GSE185477).


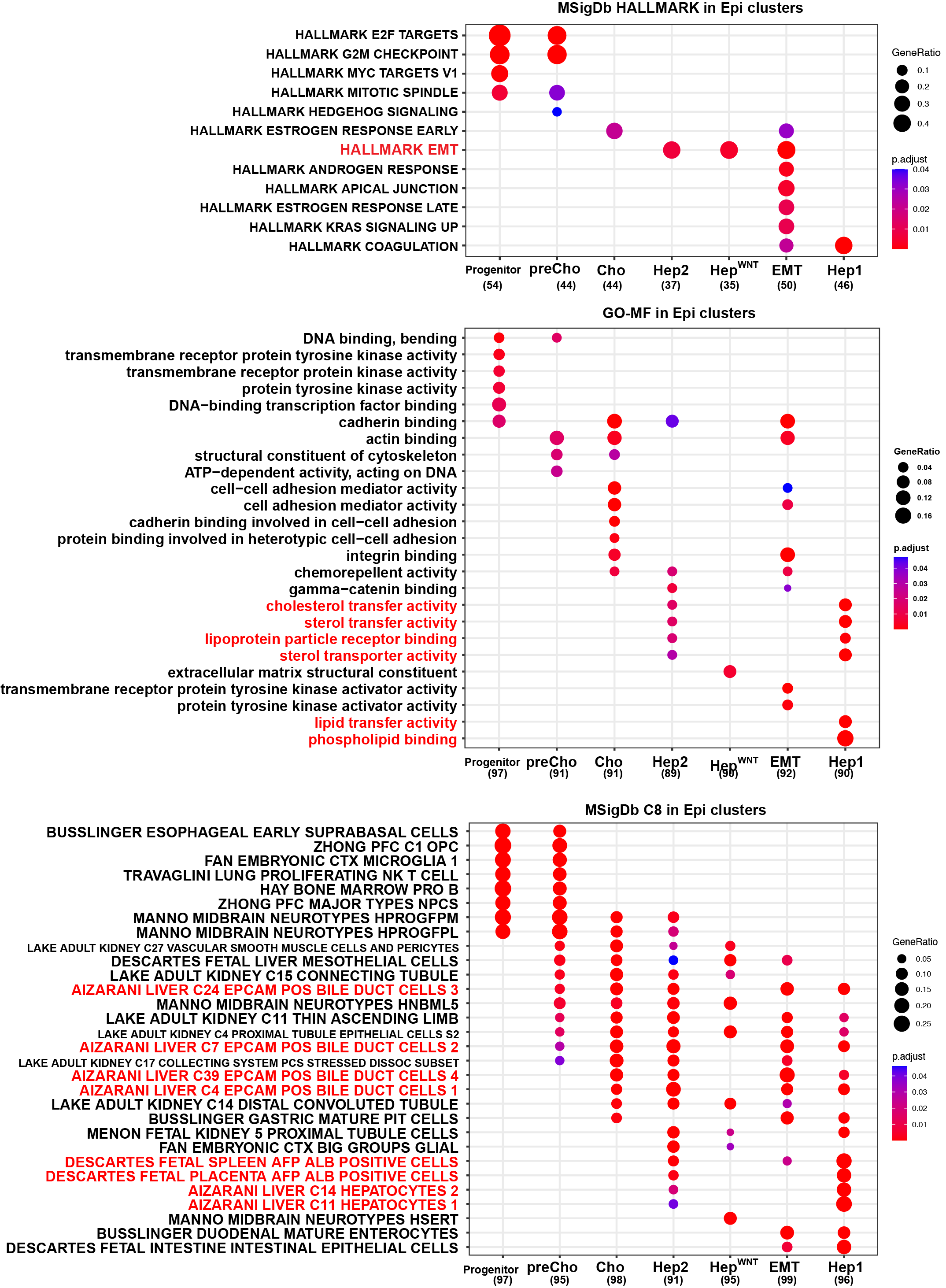


**Figure S7**. Pathway enrichment analysis for seven epithelial clusters present in microHOs. The pathways associated with the top 100 DEGs for each cell type were annotated using the MSigDb Hallmark, GO-MF and MSigDb C8 databases. The insets on the right show the gene ratio, which represents the proportion of the genes in the input gene list that are associated with a particular term; and the color-coded adjusted p-value for the statistical significance of the enrichment for a particular term in each cluster of genes.

**
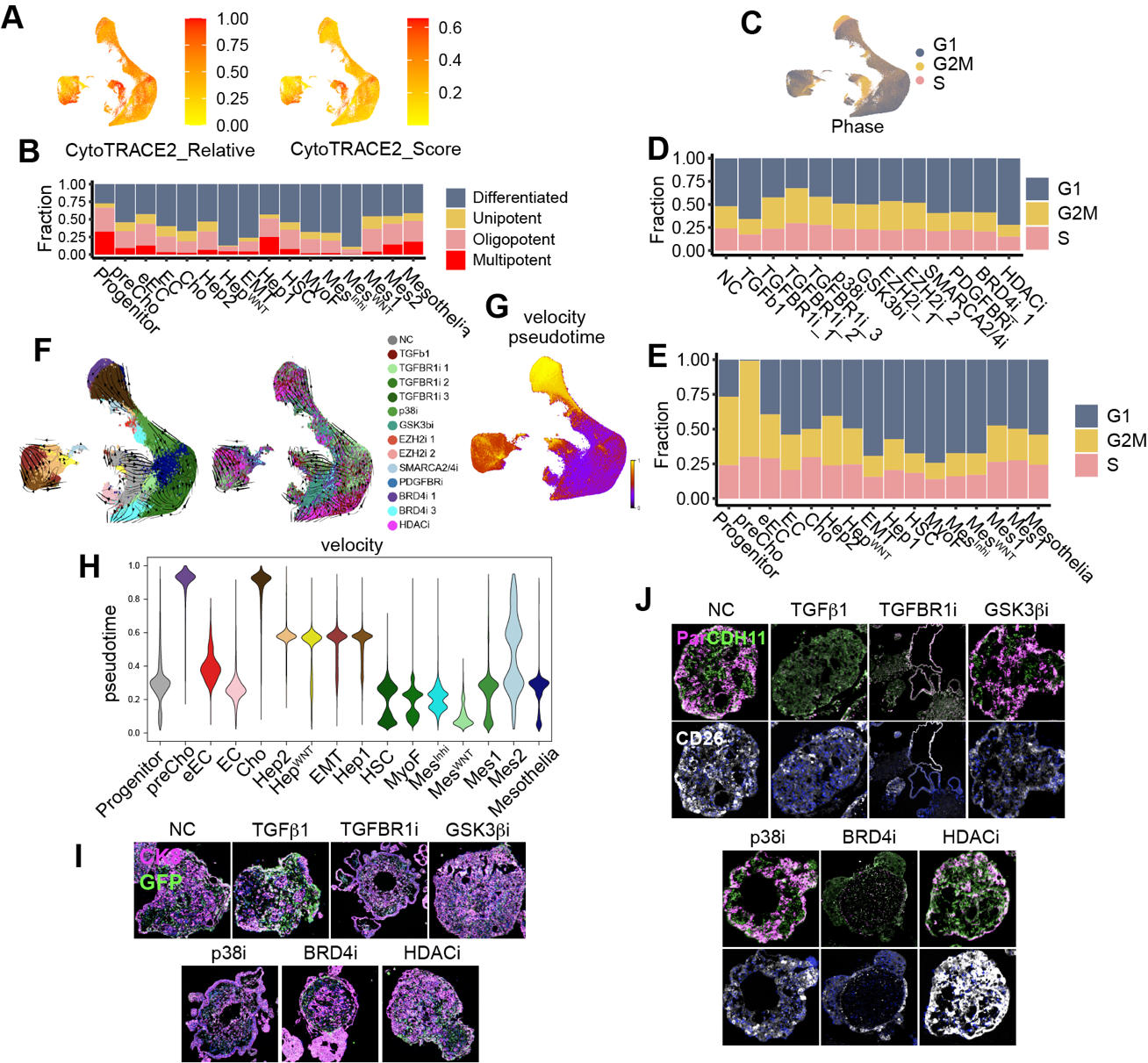
**

**Figure S8**. (**A**) Relative (left) and Score (right) are projected on UMAP from CytoTRACE2. (**B**) The ‘Cytotrace’ cell potency categories are plotted as percentage bar plot grouped by cell type in NC, TGFβ and TGFβ plus drug treated microHOs. (**C**) Cell cycle phases are projected on UMAP. (**D-E**) The cell cycle phase categories are plotted as percentage bar plot grouped by indicated treatments and cell types. (**F**) The velocity vector field displayed as streamlines of all cell type clusters (left) and treatments (right) are projected onto UMAP embedding (left). (**G**) The predicted ‘velocity_pseudotime’ are projected onto the UMAP embedding.(**H**) The predicted ‘velocity_pseudotime’ for all cell clusters in microHOs. (**I-J**) Immunostaining shows Clover^+^ (abGFP), CDH11^+^ cell population; and the CK8^+^, Par^+^ and CD26^+^ populations in microHOs receiving the indicated treatments.


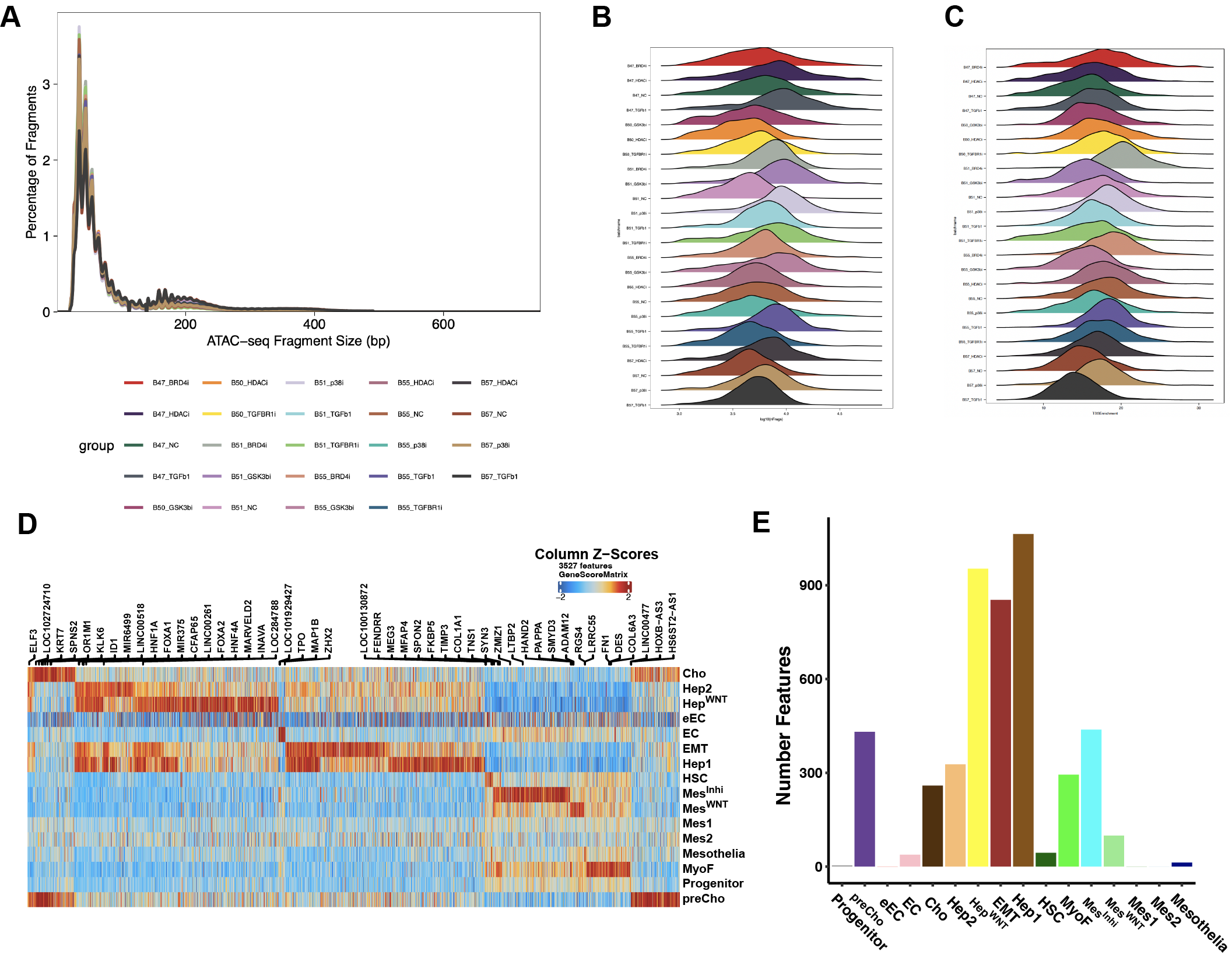


**Figure S9**. Quality control information for the ATAC-seq data. The panels show the Fragment length (**A**), the (log_10_ transformed) number of fragments (**B**), and TSS enrichment scores (**C**) in each microHO sample analyzed. **(D**) A heatmap showing the z-score scaled ‘differential gene score’ for 3527 features calculated from the snATAC-seq data, which are organized by the 16 cell types that were identified using the scRNA-seq data. (**E**) A bar plot showing the number of features identified in (C) for each of the 16 cell types.
