# Supplementary material for: AI‐Assisted Drug Re‐Purposing for Human Liver Fibrosis
Source: Adv Sci (Weinh). 2025 Sep 14;12(44):e08751. doi: 10.1002/advs.202508751 (PMC12667525; doi:10.1002/advs.202508751)
Supplement: Supplementary file 3 — Supporting Information [file ADVS-12-e08751-s002.zip › Revised Sup Data File 1.docx]

**Med-Gemini Output for Role of Epigenetic Changes in Liver Fibrosis**

**Example experiment 1** (score=novelty+details+usefulness+pairwise rank=11):

**## Prompt: A Novel Hypothesis Regarding Myofibroblast Generation in Liver Fibrosis**

We propose a novel hypothesis that specific epigenetic alterations contribute to myofibroblast (MyoF) formation in liver fibrosis. Unlike previous approaches that primarily focused on canonical TGFβ1 and PDGF pathways, we hypothesize that changes in the cell's epigenetic landscape play a critical role in regulating MyoF differentiation and subsequent ECM production.

Specifically, our hypothesis centers on the following key elements:

* **Histone Modifications:** We theorize that during fibrogenesis, alterations in histone modifications, particularly deacetylation, in the promoter regions of genes responsible for MyoF differentiation occur. For example, reduced acetylation of Histone 3 lysine 9 (H3K9ac) could promote the recruitment of repressive transcriptional machinery, silencing pro-MyoF factors. Alternatively, increased acetylation on certain histones might facilitate the binding of transcription factors that drive MyoF identity.

* **DNA Methylation:** Our hypothesis also posits that dynamic changes in DNA methylation patterns around genes involved in the MyoF pathway could be crucial. For instance, we predict that demethylation of CpG islands near regulatory elements of pro-MyoF genes might lead to enhanced transcription. Conversely, hypermethylation of the same regions might prevent the binding of critical activators, thereby inhibiting MyoF transition.

These two mechanisms, working in concert, could provide a complex regulatory system governing MyoF generation and subsequent ECM deposition impacting liver fibrosis.

**## Groundbreaking Experiment to Test the Hypothesis**

To test our hypothesis, we propose an innovative experiment utilizing the human micro-HO system:

Experiment Setup:

1. **micro-HO Generation:**

* Human micro-HOs will be generated from iPSCs according to the established protocol. These micro-HOs faithfully recapitulate the architecture and cellular composition of the human liver, including hepatic stellate cells (HSCs).

2. **Fibrosis Inducement:**

* micro-HOs will be exposed to a relevant fibrotic stimulus (e.g., TGFβ1 or PDGF) to induce the fibrotic response and MyoF generation.

* A parallel group will be kept untreated as controls.

3. **Epigenetic Profiling:**

* **ChIP-Seq**: At different time points during the fibrosis induction process, we will perform chromatin immunoprecipitation with sequencing (ChIP-Seq) to map the genome-wide landscape of relevant histone modifications such as H3K9ac, and relevant histone methylases/demethylases.

* **Methylation analysis**: Using bisulfite sequencing or similar approaches, we will assess the DNA methylation status of critical MyoF-associated genes.

4. **Candidate Identification**:

* The epigenomic analysis will guide the identification of regions with significant epigenetic changes during fibrosis development.

* Bioinformatic analysis will be conducted to correlate the epigenetic alterations with candidate genes involved in MyoF differentiation.

5. **Functional Validation:**

* The epigenetic modifications and associated candidate genes will be validated using targeted approaches. This includes:

* **CRISPR/Cas9 Epigenetic Editing:** To directly confirm the role of specific epigenetic changes, we will manipulate the epigenome using CRISPR/Cas9-based tools directly or by employing dCas9 systems fused to epigenetic writer/eraser domains.

* **Pharmacological Modulation:** To assess the therapeutic potential, pharmacological targeting of identified epigenetic modifiers will be performed. **Drugs like inhibitors of** **DNA methyltransferases** **(e.g., Azacitidine) and histone deacetylase (HDAC) inhibitors (e.g., Vorinostat) could be tested for their impact on MyoF generation and ECM production in the micro-HO model.**

**Expected Outcomes:**

* Identification of key transcriptional regulators with specific epigenetic alterations driving MyoF formation and liver fibrosis.

* Validation of epigenetic markers as new diagnostic tools for liver fibrosis.

* Discovery of potential therapeutic targets through specific epigenetic modifiers.

**Additional Techniques:**

* **Transcriptomic Analysis:** Alongside epigenomic data, RNA sequencing will be performed throughout the experiment to track the dynamics of gene expression during the fibrotic process.

* **Live Cell MyoF Imaging:** The micro-HO platform, in combination with collagen-specific fluorescent probes and second harmonic generation (SHG) microscopy, will allow for real-time live cell monitoring of MyoF emergence and ECM generation, providing invaluable insights into the kinetics of fibrosis progression.

**## Judgments and Discussion**

**Novelty and Impact**:

This hypothesis and experiment hold significant novelty as they depart from a sole focus on signaling pathways and explore the role of epigenetic mechanisms in MyoF development. The human micro-HO model, alongside powerful techniques like ChIP-Seq and live cell imaging, provide unprecedented access to a detailed understanding of fibrogenesis in a human context. This research could have a profound impact on liver fibrosis research and therapeutic advancement.

**Uniqueness of this Approach:**

Investigating epigenetic modifications in MyoF generation during liver fibrosis hasn't been extensively explored previously. While some studies investigated epigenetic changes related to HSC activation, the comprehensive and dynamic analysis we propose using micro-HOs is a significant step forward. This approach holds high potential for a deeper understanding of a complex and crucial cellular phenomenon in liver fibrosis.

**Example experiment #2** (score=novelty+details+usefulness+pairwise rank=11):

## Novel Hypothesis Regarding Myofibroblast Differentiation in Liver Fibrosis:

We hypothesize that specific epigenetic alterations, in addition to known growth factor signaling, are essential for driving the transdifferentiation of hepatic stellate cells (HSCs) into myofibroblasts – the primary collagen-producing cells responsible for fibrosis. Specifically, we propose that activation of HSCs during liver injury triggers changes in DNA methylation patterns and histone modifications at critical gene loci. These epigenetic modifications promote the expression of genes involved in:

* **Collagen production and ECM remodeling:** Genes like `COL1A1`, `COL3A1`, and matrix metalloproteinases (MMPs).

* **Myofibroblast phenotype acquisition:** Transcription factors like `SNAI1`, `TWIST1`, and smooth muscle actin (αSMA).

* **Fibrotic signaling pathways**: Genes encoding components of TGFβ, PDGF, and Hedgehog pathways.

Furthermore, based on our preliminary scRNA-seq data from ARPKD hepatic organoids, we posit that these epigenetic modifications might be driven, in part, by the STAT3 signaling pathway which is downstream of PDGFRβ activation.

## Experiment Targeting the Epigenetic Landscape :

**Objective:** To determine if epigenetic modifiers can block myofibroblast transdifferentiation and reduce collagen production in the microHO live-cell fibrosis model.

**Experimental Setup:**

1. **MicroHO Preparation:**

* Hepatic organoids will be generated from human iPSCs and differentiated into microHOs using established protocols.

* To mimic chronic liver injury, microHOs will be exposed to low, continuous doses of TGFβ1 and/or PDGF for a defined period (e.g., 7 days).

2. **Epigenetic Modifier Treatment:**

* Based on prior research and known mechanisms, we will select various classes of epigenetic modifiers for testing, including:

* **DNA Methyltransferase Inhibitors (DNMTis):** Examples include 5-Azacytidine and Decitabine, which deplete DNA methylation and reactivate silenced genes.

* **Histone Deacetylase Inhibitors (HDACis):** Examples like Trichostatin A (TSA) and Vorinostat increase histone acetylation leading to a more open chromatin state and favoring gene expression.

* **Bromodomain Inhibitors:** Such as JQ1, targeting epigenetic readers that recognize acetylated histones, thereby disrupting transcription.

3. **Assessment of Myofibroblast Transdifferentiation:**

* **Live-cell imaging**: microHOs will be monitored daily for the emergence of collagen-producing cells utilizing our established live-cell imaging system.

* **Second Harmonic Generation (SHG) microscopy:** Visualization of thick collagen fiber formation as a hallmark of fibrosis will be conducted.

* **Immunofluorescence staining:** Quantification of myofibroblast markers like αSMA and Col1A1 expression in fixed microHOs.

* **scRNA-seq analysis:** Global transcriptomic changes upon epigenetic modifier treatment will be evaluated to assess:

* Alterations in the myofibroblast gene expression signature.

* Expression changes in TGFβ, PDGF, and STAT3 pathway components

* Identification of specific genes activated or repressed by epigenetic modifications.

4. **Evaluation of Collagen Production and ECM deposition:**

* **Quantitative analysis of collagen content:** Utilizing biochemical assays like Sircol assay or hydroxyproline quantification.

* **Measurement of MMP activity:** Assays will assess the ECM remodeling dynamics influenced by epigenetic modifications.

**Expected Outcome and Impact:**

This experiment has the potential to:

* Uncover novel epigenetic changes driving myofibroblast transdifferentiation and collagen production in liver fibrosis.

* Determine the efficacy of epigenetic modifiers in reducing or reversing hepatic fibrosis in the microHO model.

* Identify promising drug targets within the epigenetic landscape of fibrosis.

* Provide a valuable platform for drug discovery and validation for liver fibrosis treatments.

**Novelty and Significance:**

* **Innovative Hypothesis:** Linking specific epigenetic changes to myofibroblast transdifferentiation represents a novel departure from the prevailing focus solely on growth factor signaling.

* **Advanced Experimental Platform:** The utilization of human microHOs offers a physiologically relevant and species-specific model system to study fibrosis.

* **Detailed Experimental Design:** The multi-faceted approach employing live-cell imaging, molecular biology, and transcriptomics provides a comprehensive analysis of myofibroblast behavior and collagen deposition.

**Similarity assessment:**

While studies have explored epigenetic factors in broad cellular models of fibrosis, this experiment's strength lies in its specific focus on myofibroblast generation and collagen production within the context of the innovative microHO liver fibrosis model. This detailed approach encompassing multiple analytical techniques provides a more nuanced understanding of the epigenetic landscape. While other groups may explore aspects of epigenetics in fibrosis, the proposed combination of hypothesis, methodology, and model system likely distinguishes it from existing research.
